# Adding highly variable genes to spatially variable genes can improve cell type clustering performance in spatial transcriptomics data

**DOI:** 10.1093/bioadv/vbaf285

**Published:** 2025-11-20

**Authors:** Yijun Li, Stefan Stanojevic, Bing He, Zheng Jing, Qianhui Huang, Jian Kang, Lana X Garmire

**Affiliations:** Department of Biostatistics, University of Michigan, Ann Arbor, MI 48109, United States; Department of Computational Medicine and Bioinformatics, University of Michigan, Ann Arbor, MI 48105, United States; Department of Computational Medicine and Bioinformatics, University of Michigan, Ann Arbor, MI 48105, United States; Department of Applied Statistics, University of Michigan, Ann Arbor, MI 48109, United States; Department of Computational Medicine and Bioinformatics, University of Michigan, Ann Arbor, MI 48105, United States; Department of Biostatistics, University of Michigan, Ann Arbor, MI 48109, United States; Department of Biostatistics, University of Michigan, Ann Arbor, MI 48109, United States; Department of Computational Medicine and Bioinformatics, University of Michigan, Ann Arbor, MI 48105, United States

## Abstract

**Motivation:**

Spatial transcriptomics has allowed researchers to analyze transcriptome data in its tissue sample’s spatial context. Various methods have been developed for detecting spatially variable genes (SV genes), whose gene expression over the tissue space shows strong spatial autocorrelation. Such genes are often used to define clusters in cells or spots downstream. However, highly variable (HV) genes, whose quantitative gene expressions show significant variation from cell to cell, are conventionally used in clustering analyses.

**Results:**

In this report, we investigate whether adding highly variable genes to spatially variable genes can improve the cell type clustering performance in spatial transcriptomics data. We tested the clustering performance of HV genes, SV genes, and the union of both gene sets (concatenation) on over 50 real spatial transcriptomics datasets across multiple platforms, using a variety of spatial and non-spatial metrics. Our results show that combining HV genes and SV genes can improve overall cell-type clustering performance.

**Availability and implementation:**

All data and code used in this evaluation study can be found in the following link: https://github.com/lanagarmire/ST_benchmark.

## 1 Introduction

Spatial omics technologies are one of the breakthroughs in science in the last several years (Liu *et al.* 2020, Rao *et al.* 2021, Deng *et al.* 2022, Zhang *et al.* 2023). Such technologies are able to systematically measure transcriptome information in the tissue space, thereby preserving the spatial context of gene expression. The addition of spatial information allows researchers to further explore biological architecture and function and reveal more insights with respect to various disease mechanisms (Lee *et al.* 2017, Moncada *et al.* 2020, Hunter *et al.* 2021, Takei *et al.* 2021). Many techniques for sequencing spatially resolved transcriptome data have been developed, including MERFISH (Moffitt *et al.* 2018, Xia *et al.* 2019), Visium (Ståhl *et al.* 2016, Thrane *et al.* 2018), as well as the more recent platforms at the single-cell resolution such a**s** cosMx SMI (He *et al.* 2021, Moffet *et al.* 2023, Sankowski *et al.* 2024), Xenium (Chen *et al.* 2020, Floriddia *et al.* 2020). Such technologies can be categorized into two general classes: fluorescence in situ hybridization (FISH)-based methods such as MERFISH, cosMx, and Xenium, which directly extract transcriptome information at a molecular level and obtain the spatial locations of the cells through imaging techniques; and Next Generation Sequencing (NGS)-based methods such as Visium, which attach probes with fixed physical locations to cryosections of tissues to obtain transcriptome information.

Many interesting features can be extracted from spatial transcriptomics data for downstream functional analysis, including spatially variable (SV) genes and highly variable (HV) genes. SV genes are unique features of spatial transcriptomics data due to the added spatial context. The expression of an SV gene shows distinct spatial autocorrelation. Such properties are indicative of the partition of the spots or cells (Edsgärd *et al.* 2018, Svensson *et al.* 2018, Sun *et al.* 2020, Dries *et al.* 2021, Miller *et al.* 2021). In the spatial transcriptomics literature, the clustering of spatial transcriptomics datasets usually refers to defining spatial domains (Hu *et al.* 2021, Dong and Zhang 2022, Xu *et al.* 2022). The role of spatially variable genes in clustering cell types or the spot-level cellular composition profile, however, remains relatively uninvestigated. HV genes, on the other hand, are genes whose expression values significantly vary without considering the constraints of the spots’ physical locations. HV genes are conventionally used for clustering analysis to group cells with similar gene expression profiles. Notably, SV genes and HV genes are often distinct feature sets in spatial transcriptomics data (see Supplementary Fig. 1 and Tables 1–2), despite some gene overlap. As a result, clustering based on SV genes or HV genes alone may yield biases for downstream functional annotations.

We therefore asked if adding the conventional HV genes to the SV genes can reveal more biological insights and help to improve cell-type clustering performance of spatial transcriptomics data, an area currently unexplored. Towards this goal, we benchmarked the downstream clustering performance of several gene sets: HV genes, SV genes, and the union of HV and SV genes. We tested over 50 ST datasets across 4 ST platforms of single-cell or spot resolutions, including Vizgen’s MERFISH, Nanostring’s cosMx, and 10X Genomics’ Visium and Xenium, and evaluated the results using a comprehensive set of metrics. Our results show that adding HV to SV genes can help improve clustering performance and reveal more biological insights for downstream analysis.

## 2 Methods

### 2.1 Ground truths for real data sets

The quality of the ground truth labels is essential to the evaluation of the methods’ performance. For the real datasets, we obtained ground truth labels from the original studies (see Supplementary Table 4). The ground truth labels were obtained through either manual supervised annotation with scRNA-seq references or through supervised cell segmentation using platform-specific topological data (for some Xenium datasets and cosMx datasets). The ground truth labels are validated in the original studies and are therefore suitable for our benchmark study.

### 2.2 Real datasets and preprocessing

The present study utilized a set of 51 real datasets to account for potential confounding effects arising from a range of factors, including technology platform, resolution, tissue type, and clinical phenotype. To ensure a comprehensive representation of major current Spatial Transcriptomics platforms, we chose 10 datasets from Visium, including a Mouse Olfactory Bulb study (Ståhl *et al.* 2016), an Ovarian Cancer study (Sanders *et al.* 2022), and a Breast Cancer study (Andersson *et al.* 2021). We also included 12 datasets from MERFISH (Vizgen) on the Mouse Brain Hypothalamic region (Moffitt *et al.* 2018), 10 datasets from Xenium on the human kidney (Benjamin *et al.* 2024) and Mouse Brain Anterior Thalamic Nuclei (ATN) (Kapustina *et al.* 2024), and 20 datasets from CosMx (Nanostring) on human Non-Small Cell Lung Cancer (NSCLC) (He *et al.* 2022). The Visium dataset provides non-single-cell resolution, whereas the remaining datasets offer single-cell resolution. The datasets cover a diverse range of tissue types and disease types, allowing for robust and comprehensive analysis.

Before extracting the HV and SV genes, we preprocessed the data by first filtering the raw gene expression dataset. In order not to over-filter the data before analysis, we computed the average expression amongst the genes and the cells and removed those that were statistical outliers. Furthermore, we removed small population cell types that took up less than 5% of the entire cell population to avoid potential bias in rare cell type annotations and class imbalance bias for external clustering validation. For the MERFISH datasets specifically, we rescaled the raw data by a factor of 1000, similar to the preprocessing steps in the SPARK paper (Sun *et al.* 2020). For data normalization, we used log-normalization for the MERFISH datasets. For the remaining datasets, we normalized the data using the method developed by Lause *et al.* (Lause *et al.* 2021) and performed downstream dimension reduction analysis on the Pearson residuals.

### 2.3 Selection of HV and SV genes

For MERFISH datasets, we used a LOESS regression model with each gene’s log mean expression as the independent variable and the coefficient of variance as the dependent variable. We obtain the difference between the genes’ predicted coefficient of variance and their actual coefficient of variance values. We retain genes whose difference in coefficient of variance is larger than zero. We set the following three thresholds for HV genes in the MERFISH datasets: the 50th percentile for the low threshold, the 70th percentile for the medium threshold, and the 90th percentile for the high threshold. For the remaining datasets, we obtained the HV genes by looking at the genes whose Pearson residual variance of the normalized gene expression is larger than 1. Similar to the MERFISH datasets, we set the low threshold at the 50th percentile, the medium threshold at the 70th percentile, and the high threshold at the 90th percentile.

We used SPARK to detect SV genes. We set the low threshold at the common *P*-value cutoff at .05. We used an additional custom procedure to further threshold SV genes. For the medium threshold level, we set the p-value cutoff at the 25th percentile. For the high threshold level, we set the p-value threshold at the 50th percentile. See Supplementary Tables 1–2 for details on the number of HV genes, SV genes, and concatenation genes for each dataset. For the concatenation gene sets, we remove any potential duplicated genes that are both highly variable and spatially variable so that each gene appears in the concatenation gene set at most once. To test robustness, we used LOESS smoothing as an additional HV genes detection approach and spatialDE as an additional SV genes detection approach. The cutoff thresholds for both gene sets remained the same as Pearson residuals and SPARK.

### 2.4 Clustering methods

We focus on results generated via Leiden clustering on shared nearest networks, a commonly used approach in Spatial Transcriptomics analysis (Traag *et al.* 2019). We further validated our results using multiple additional common clustering methods for transcriptomics data, including kmeans clustering (Lloyd, 1982), Monocle3 (Cao *et al.* 2019), cellTree (duVerle *et al.* 2016), and SC3 (Kiselev *et al.* 2017).

**Leiden** (Traag *et al.* 2019) is a community detection clustering algorithm. Using the principal components of each dataset and gene set, Leiden builds a shared nearest neighbor network and clusters the cells based on the connectivity information in said network. We tuned the resolution parameter using a grid search strategy. At each attempted resolution value, we repeatedly run Leiden clustering 10 times under different random seeds. We keep the resolution parameters corresponding to the same number of clusters as there are in the ground truth labels, and select the final clustering labels by picking the majority set. The number of nearest neighbors for building the network is set to 15 for all datasets. We used the Euclidean distance between the principal components to compute unsupervised clustering metrics for Leiden.

**Kmeans** (Lloyd, 1982) is a very common clustering algorithm that partitions the cells into a predefined number of clusters with the nearest centroid. We set the predefined number of clusters to the number of ground truth clusters. We performed kmeans clustering on the selected principal components of each dataset and gene set. We explored three different distance measures: Euclidean distance, Pearson correlation, and Spearman correlation. These measures were directly used to compute the unsupervised clustering metric, Pearson Gamma Coefficient, for kmeans.

**Monocle3** (Cao *et al.* 2019) also uses a community detection algorithm. Instead of a shared nearest neighbor network, Monocle3 uses a k-nearest neighbor network. We also tuned and selected the optimal resolution parameter using the same optimization strategy employed for Leiden. The number of nearest neighbors for building the network is set to 15 for all datasets. We used the cosine distance between the principal components to compute the unsupervised clustering metric for Monocle3.**cellTree** (duVerle *et al.* 2016) is a clustering algorithm originally developed for scRNA-seq data. cellTree uses Latent Dirichl*et al*location (LDA) to model single-cell data. The fitted LDA model is composed of a set of topic distributions for each cell and per-topic gene distributions. Per-cell topic histograms can then be used as a low-dimensional embedding to evaluate cell similarity and infer hierarchical relationships, while analysis of the topics themselves can provide useful biological insights on the sets of genes driving the different stages of the process studied. The result of cellTree contains the empirical topic probability per cell. By extracting the maximum probability topic, we can assign cluster labels. We use the raw gene expression for each respective gene set as input, and set the number of topics (clusters) to the number of ground truth clusters. We further use the cosine distance between the topic distribution of the cells to compute the unsupervised clustering metric, Pearson Gamma for cellTree.

**SC3** (Kiselev *et al.* 2017) clusters cells via consensus clustering. SC3 runs kmeans under a combination of distance (Euclidean, Pearson correlation, and Spearman correlation) and dimension reduction (PCA, graph laplacian) strategies. SC3 then computes a consensus matrix measuring the similarity between each set of clustering labels using a Cluster-based similarity partitioning algorithm (CSPA). Finally, SC3 performs hierarchical clustering on the consensus matrix using Euclidean distance to obtain the final consensus cluster labels. We used the Euclidean distance of the consensus matrix to compute the unsupervised clustering metric, Pearson Gamma for SC3.

### 2.5 Evaluation metrics

**Adjusted Mutual Information (AMI)**: To evaluate the general accuracy of clustering in spatial transcriptomics data, we computed the Adjusted Mutual Information (AMI) (Vinh *et al.* 2010) of each set of clustering results compared with the ground truth. AMI measures the level of concordance between two sets of labels and is widely used for measuring clustering accuracy in scRNA-Seq datasets with no spatial context (Peyvandipour *et al.* 2020, Wang *et al.* n.d; Li *et al.* 2020). AMI is bounded between 0 and 1, with higher values indicating better clustering performance.

**Weighted F1**: To evaluate clustering accuracy while accounting for the specific classification accuracy of each cluster/cell type, we used weighted F1. We match the clustering labels with the ground truth labels via cluster/cell-type-specific average gene expression. Then we computed the F1 score for each cluster and obtained a weighted F1 by computing the average of cluster-specific F1 scores, weighted by the cluster sample size.

**Pearson Gamma**: To evaluate the consistency of clustering results, we also computed the Pearson Gamma coefficient of the clustering labels, which measures the correlation between the pairwise distance between data points and their cluster memberships. Specifically, we compute a binary membership matrix where an entry is 1 if the respective data points are assigned the same label and 0 otherwise. The correlation between such pairwise membership and the pairwise distances is computed as the Pearson Gamma coefficient.

**Spatial Concordance (SC)**: To evaluate the clustering accuracy in the spatial context, we develop a metric that takes into account the local spatial heterogeneity of cell types, which we denote as spatial concordance (SC). The larger the SC is, the more accurate spatial clustering is compared to the ground truth. We define the local spatial neighborhood as a square with side length 2d, where d is chosen to be the top 1% of the cell (or spot) pairwise distances on the tissue sample. Furthermore, we denote the set of ground truth labels as u and the set of clustering labels of a certain computational strategy as $u^{*}$ (assuming the labels in $u^{*}$ have been matched to the ones in u). For each spot i, we compute the entropy of ground truth labels within its local spatial neighborhood, denoted as $e_{i}$. The higher $e_{i}$ is, the more heterogeneous the neighborhood of i. The set of local entropy values is then standardized as below, denoted as $e_{i}^{*}$.


$$e_{i}^{*}=e_{i}/\sum_{i}e_{i}$$

The spatial concordance is computed as below, where a match in a more heterogeneous neighborhood is weighed higher than one in a relatively homogeneous neighborhood, prioritizing edge cases on domain borders.


$$\sum_{i}I(u_{i}==u_{i}^{*})\cdot e_{i}$$

**Mean Spatial AMI**: Similar to Spatial Concordance, we compute the AMI of the local neighborhood of each spot i. Then, we compute the mean spatial AMI by computing the average spatial AMI weighted by the entropy of each spot/cell.

### 2.6 Hypothesis testing

Given our sample sizes are relatively small for each platform and that the metrics are not necessarily normally distributed, we performed paired Wilcoxon signed-rank tests on the evaluation metrics for different gene sets in order to assess the robustness of the clustering performance.

## 3 Results

### 3.1 Overview of computational workflow

**Figure 1.**
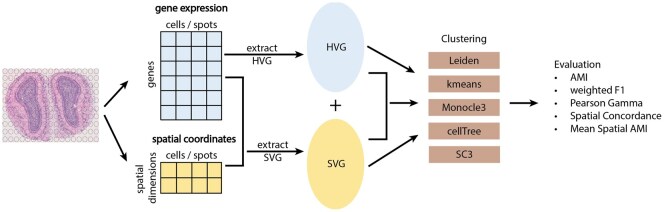
Study workflow. The workflow is composed of four general steps. Step 1: Extraction of the HV and SV genes. Step 2: Add the HV genes to the SV genes. Step 3: perform cell-type clustering analysis on HV genes, SV genes, and the union of SV and HV genes. Step 4: Evaluate the cell-type clustering performance using non-spatial and spatial clustering metrics.

The workflow for this study is shown in Fig. 1. It starts with spatial transcriptomics data, which has two components: a gene expression matrix and spatial data, which consists of the spatial coordinates of each spot or cell. After data preprocessing (see Methods), we extracted the HV genes using the gene expression matrix and the SV genes using both gene expression and spatial coordinates. We use Leiden clustering, a community detection-based clustering method commonly used for clustering transcriptomics data, as our default clustering method (Traag *et al.* 2019, Heumos *et al.* 2023). Since our main interest is to cluster based on the expression profiles of different gene sets, Leiden clustering is a very suitable option. For the gene sets: HV genes, SV genes, and concatenation (union set of HV and SV genes), we reduced the feature dimensions using Principal Component Analysis (PCA). We then constructed shared nearest neighbor networks (sNN) using the top Principal Components (PCs) and performed Leiden clustering on the sNN. Besides Leiden clustering, we also investigated other methods for clustering gene expression profiles, such as kmeans clustering (Lloyd, 1982), Monocle3 (Cao *et al.* 2019), cellTree (duVerle *et al.* 2016), and SC3 (Kiselev *et al.* 2017) (see Supplementary Table 3). We analyzed the clustering performance using supervised metrics such as AMI and weighted F1 scores across clusters, unsupervised metric Pearson Gamma coefficients, and supervised spatial metrics such as Spatial Concordance (SC) and mean Spatial AMI. Besides the overall clustering performance, we also examined local, cluster-specific, and spot/cell-specific metrics. We applied the above pipeline to a total of 51 real datasets across four spatial transcriptomics platforms, including Visium, Xenium, MERFISH, and CosMx.

### 3.2 Clustering accuracy on real spatial transcriptomics datasets

We compared the clustering accuracy performance of computational strategies on diverse technical platforms and tissue types (Supplementary Table 4) with matching ground truth labels (Methods). We selected datasets where the ground truth labels have been verified in the original publications. These datasets include both human and animal tissue, as well as multiple underlying conditions such as ovarian cancer, breast cancer, etc.

**Figure 2.**
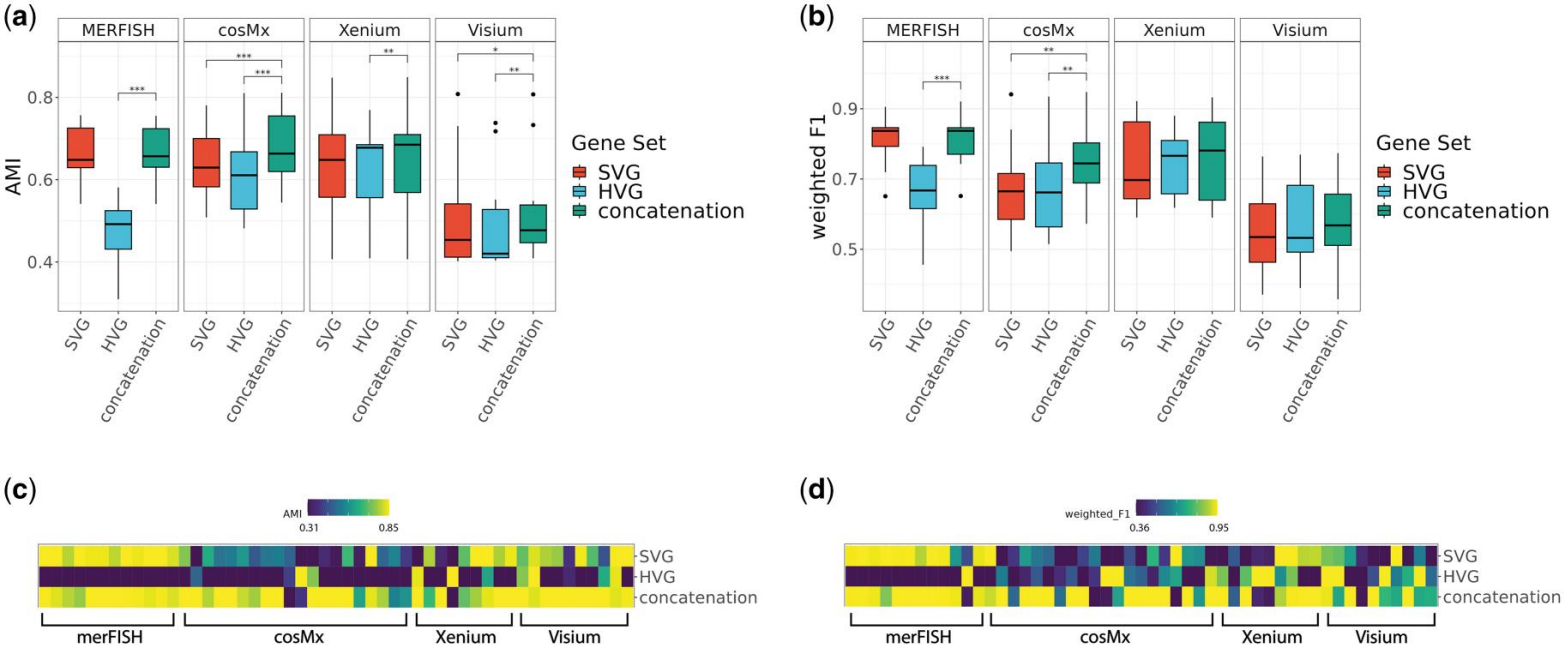
Comparisons of supervised, non-spatial clustering performance of the default clustering method (Leiden) on real spatial transcriptomics datasets, in four representative platforms including MERFISH, cosMx, Xenium, and Visium at default HV genes threshold level (low) and default SV genes threshold level (low). (a, b) boxplot of AMI and weighted F1 for 51 real datasets, divided by platform. (b, d) heatmaps of AMI and weighted F1 for all 51 real datasets, ordered by platform. ***P-value < 1e−3; **1e−3 ≤ *P*-value < 1e−2; *1e−2 ≤ *P*-value < 5e−2; 5e−2 ≤ *P*-value < .1.

**Figure 3.**
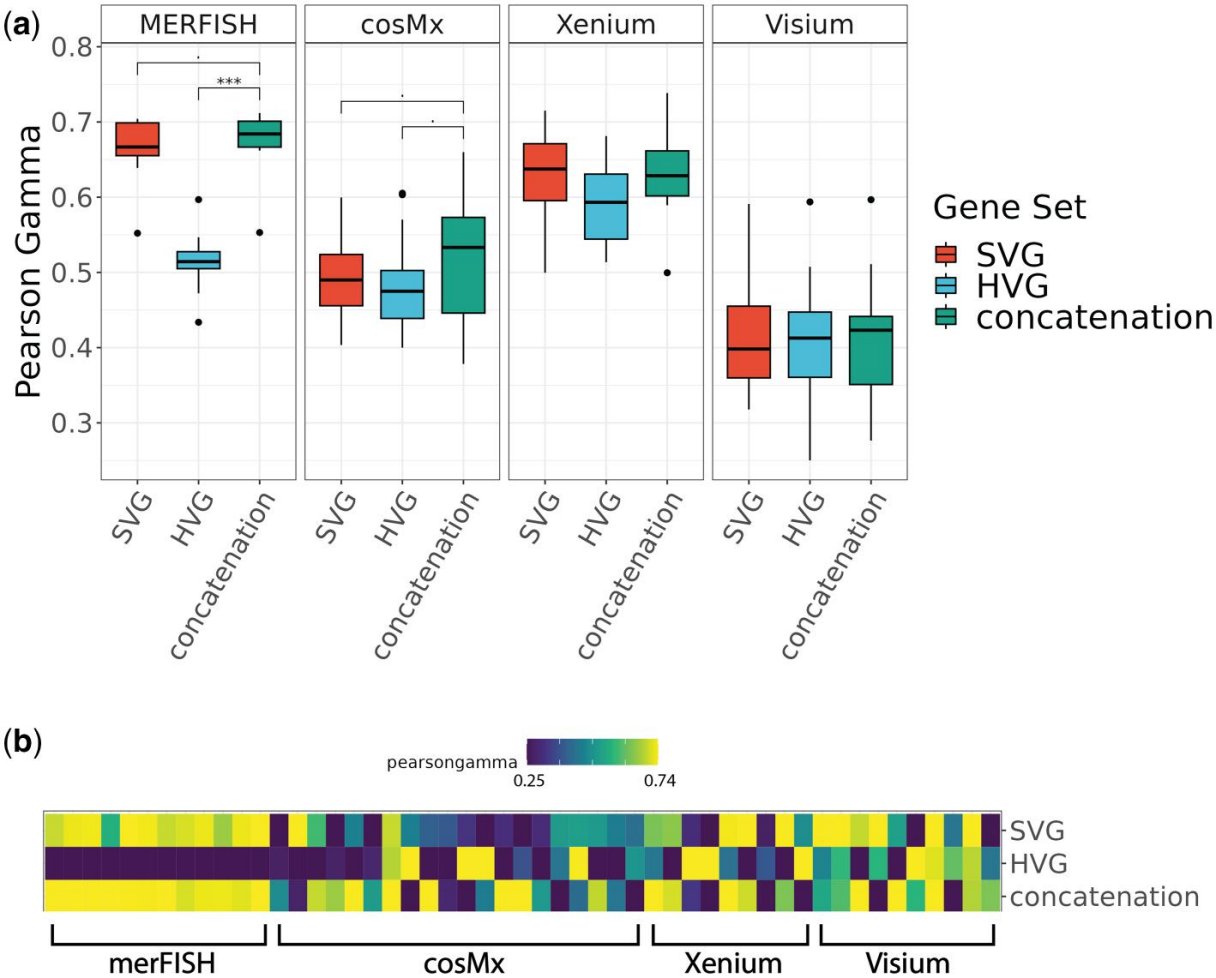
Comparisons of unsupervised, non-spatial clustering performance of the default clustering method (Leiden) on 51 real spatial transcriptomics datasets, in four representative platforms including MERFISH, cosMx, Xenium, and Visium at default HV genes threshold level (low) and default SV genes threshold level (low). (a) boxplot of Pearson Gamma, divided by platform. (b) heatmap of Pearson Gamma, ordered by platform. ***P-value < 1e−3; **1e−3 ≤ *P*-value < 1e−2; *1e−2 ≤ *P*-value < 5e−2; 5e−2 ≤ *P*-value < .1.

The accuracy of the main clustering methods of the HV genes, SV genes, and their union gene set across the real datasets is shown in Fig. 2. Concatenation of HVG and SVG consistently improved clustering performance compared to using either s*et al*one across all platforms (Fig. 2a and b, Supplementary Table 5). This trend was particularly evident in cosMx, Xenium, and Visium datasets, where concatenation generally achieved higher AMI and weighted F1 scores. Heatmaps further illustrate that these improvements are robust across individual datasets (Fig. 2c and d). To further check the robustness of concatenation’s improvement in clustering performance, we repeatedly selected random sets of genes with the same size as the concatenation gene set that were neither highly variable nor spatially variable. We deemed these random sets as the “ground control” condition. As shown in Supplementary Fig. 2a and b, clustering performance based on random gene sets was consistently worse than that of HV genes, SV genes, or their concatenation across all metrics, among probe-based and sequencing-based datasets. These results confirm that the advantage of concatenation cannot be attributed to the mere increase in gene set size, but rather reflects the complementary information captured by combining HV genes and SV genes. Similarly, we observed improvement in the unsupervised metric Pearson Gamma when combining HV and SV genes, as opposed to HV or SV genes alone (Fig. 3, Supplementary Table 5). Similar to AMI and weighted F1, random gene sets did not achieve better performance than concatenation in Pearson Gamma (see Supplementary Fig. 2c).

**Figure 4.**
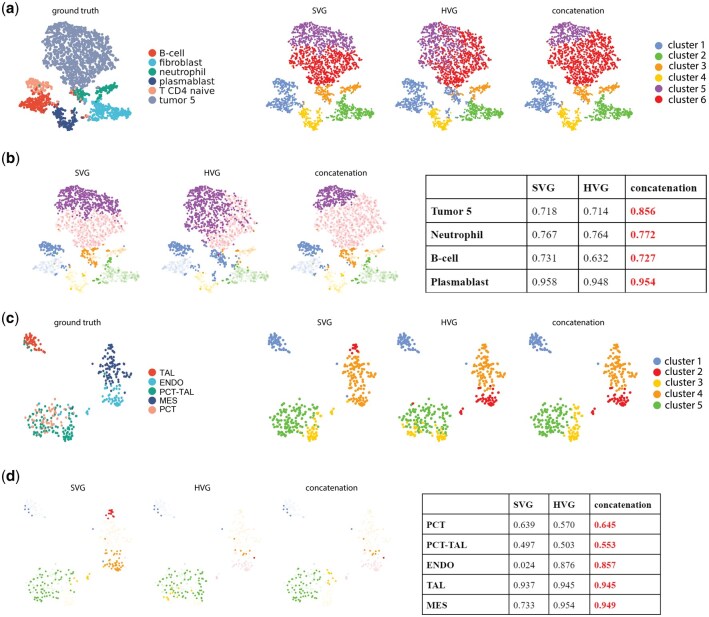
Comparison of cluster performance of SV genes, HV genes, and their union set for Leiden for representative datasets on the tSNE space for the union set. (a) Comparison of clustering labels for cosMx NSCLC dataset for patient 2, FOV 7. (b) Comparison of tSNE space highlighting mis-classified clusters for each gene set for cosMx NSCLC dataset for patient 2, FOV 7, with cluster-specific F1 scores for each gene set summarized in a table. (c) Comparison of clustering labels for Xenium Kidney dataset sample N7. (d) Comparison of tSNE space highlighting mis-classified clusters for each gene set in Xenium Kidney dataset sample N7, with cluster-specific F1 scores for each gene set summarized in a table. Abbreviations: TAL, thick ascending limb; ENDO, endothelial cells; PCT-TAL, proximal convoluted tube–thick ascending limb; MES, mesangial cell; PCT, proximal convoluted tube.

We further examined the specific advantages of combining HV genes and SV genes through a closer look at spot/cell-level clustering performance (Fig. 4, Supplementary Figs. 3 and 4). In the cosMx dataset, for patient 5–2’s field of view (FOV) 7 (Fig. 4a and b), though all three gene sets (SVG, HVG, and concatenation) struggle to separate B and T cells under the 6-cluster constraint, concatenation had the lowest error of distinguishing tumor cells, compared to SVG and HVG conditions. In another representative kidney Xenium dataset (Fig. 4c and d), combining HV genes and SV genes improved the delineation between the proximal convoluted tube (PCT) and proximal convoluted tube—thick ascending limb (PCT-TAL), as well as the classification for other cell types such as endothelial cells (ENDO), mesangial cell (MES), and thick ascending limb (TAL). Similar improvement in delineation of specific cell types in other datasets was observed, for example, combining HV genes and SV genes led to better classification of inhibitory neurons in MERFISH mouse hypothalamus data (Supplementary Fig. 3a and b), as well as better identification of cancer cells, connective tissues, and immune cells in Visium’s Breast Cancer dataset (Supplementary Fig. 3c and d).

### 3.3 Spatially adjusted clustering accuracy on real spatial transcriptomics datasets

**Figure 5.**
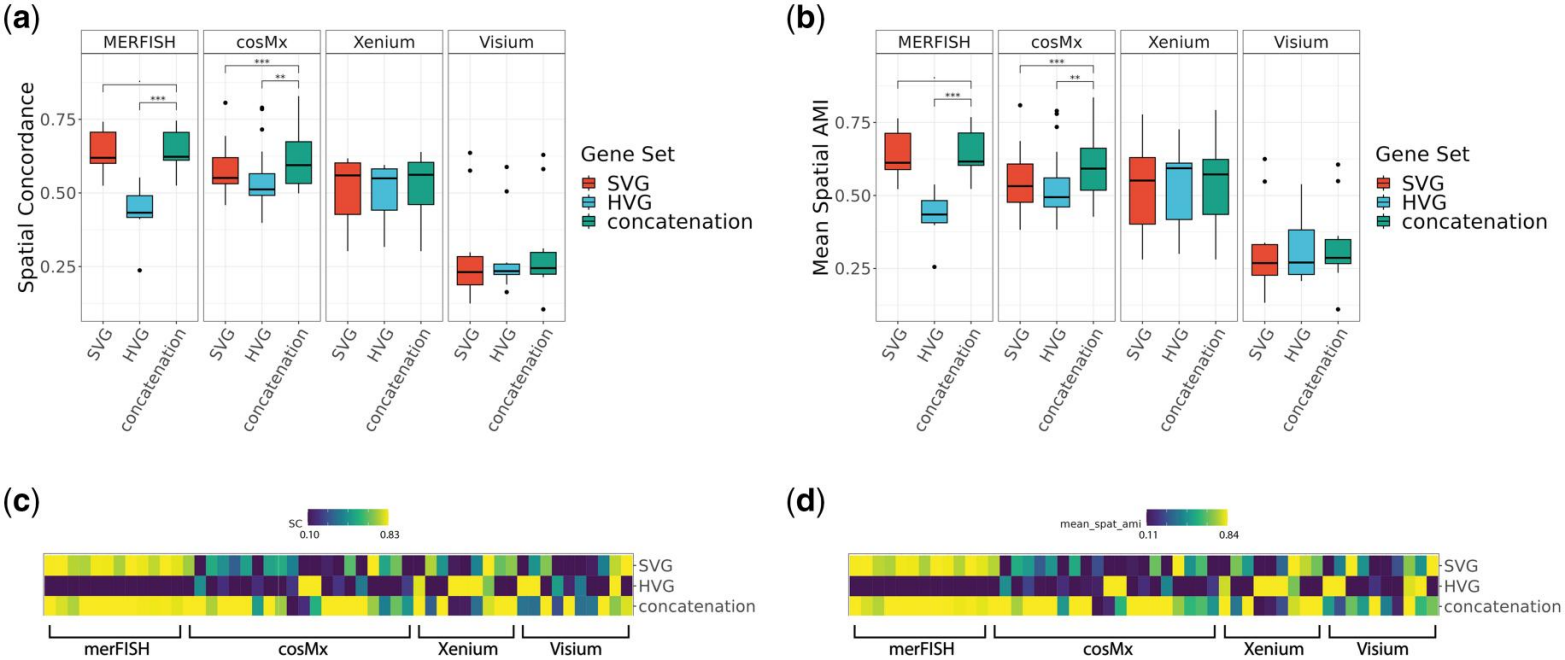
Comparisons of spatial clustering performance of the default clustering method (Leiden) on 51 real spatial transcriptomics datasets, in four representative platforms including MERFISH, cosMx, Xenium, and Visium at default HV genes threshold level (low) and default SV genes threshold level (low). (a, b) boxplots of SC (Spatial Concordance) and Mean Spatial AMI, divided by platform. (b, d) heatmaps of SC and Mean Spatial AMI, ordered by platform. ***P-value < 1e−3; **1e−3 ≤ *P*-value < 1e−2; *1e−2 ≤ *P*-value < 5e−2; 5e−2 ≤ *P*-value < .1.

Since spatial transcriptomics data measure gene expression in situ, we also evaluated the clustering performance of each computational strategy by taking into account the spatial distribution of the spots. Towards this, we derived two novel spatially adjusted clustering metrics: spatial concordance (SC) and mean spatial AMI, to measure the clustering accuracy in the spatial context (see Methods). As shown in Fig. 5, concatenation of HVG and SVG consistently improved spatial clustering performance compared to using either s*et al*one, as measured by spatial concordance and mean spatial AMI (Fig. 5a and b, Supplementary Table 5). As in conventional clustering metrics, we also observed platform-specific variations in the clustering performance of gene sets. The improvement was particularly strong in MERFISH and cosMx datasets, where concatenation yielded substantially higher scores. For Xenium and Visium, concatenation also generally outperformed or matched the single gene sets. Heatmaps demonstrate that concatenation achieves robust gains across individual datasets (Fig. 5c and d). Performance with “ground control” random genes performance (see Supplementary Fig. 2d and e) is generally worse than concatenation w.r.t. Spatial Concordance and Mean Spatial AMI.

**Figure 6.**
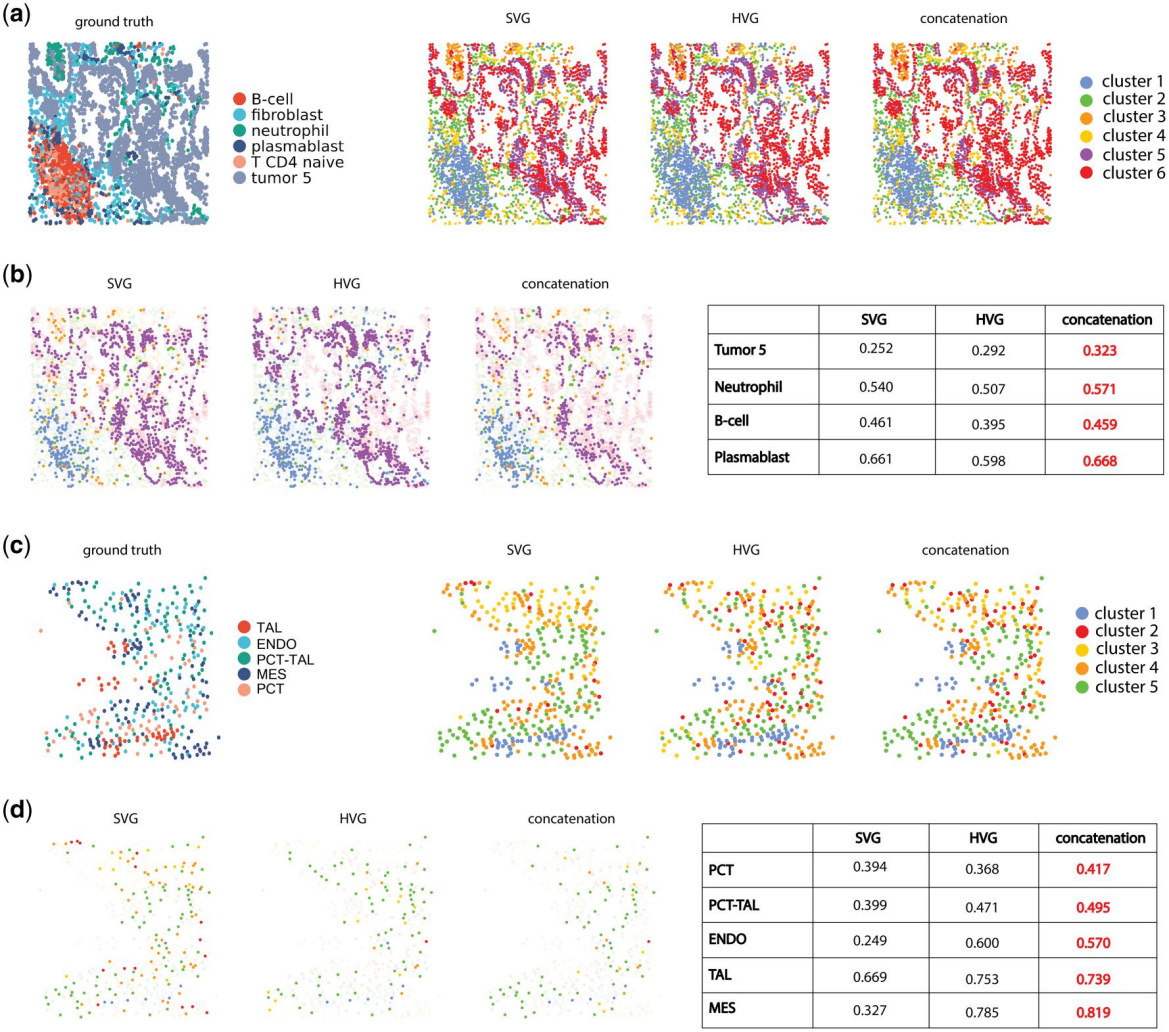
Comparison of cluster performance of SV genes, HV genes, and their union set for Leiden for representative datasets. (a) Comparison of clustering labels for cosMx NSCLC dataset for patient 2 FOV 7. (b) Comparison of tissue space highlighting mis-classified clusters for each gene set in cosMx NSCLC dataset for patient 2 FOV 7, with cluster-specific spatial AMI scores for each gene set summarized in a table. (c) Comparison of clustering labels for Xenium Kidney dataset sample N7. (d) Comparison of tissue space highlighting mis-classified clusters for each gene set in Xenium Kidney dataset sample N7, with cluster-specific spatial AMI scores for each gene set summarized in a table. Abbreviations: TAL, thick ascending limb; ENDO, endothelial cells; PCT-TAL, proximal convoluted tube–thick ascending limb; MES, mesangial cell; PCT, proximal convoluted tube.

We also examined the clustering labels and cell/spot-level spatial clustering performance (Fig. 6, Supplementary Figs. 5 and 6) in the tissue context. For the representative cosMx dataset (Fig. 6a and b), we observed that the tumor cells have distinct spatial patterns, whereas the immune cell types, such as B-cells, neutrophils, and plasmablasts, have much more subtle distributions across the tissue. Combining the HV and SV genes significantly improves the classification of tumor cells per spatial AMI metric, but not much for the other cell types. In the representative Xenium dataset (Fig. 6c), we also observed improved cluster-specific mean spatial AMI for the cell types of thick ascending limb (TAL), proximal convoluted tube (PCT), proximal convoluted tube–thick ascending limb (PCT-TAL), endothelial cells (ENDO), and mesangial cells (MES). Notably, the improvement is more striking for ENDO and MES. In addition to the coxMx and Xenium datasets, we observed similar improvements in combining HV genes and SV genes in the representative MERFISH dataset, where we observed improved mean spatial AMI in inhibitory neurons (Supplementary Fig. 5b). Similarly, in the representative Visium dataset, we observed improved mean spatial AMI in cancer cells, connective tissues, and immune cells (Supplementary Fig. 5d).

### 3.4 The effect of clustering method and gene set selection threshold

To further validate our findings, we also examined how our conclusion is affected by different clustering methods, by the stringency thresholds of HV and SV gene selection. Besides Leiden clustering, whose results are featured in the main figures, we also performed other clustering analyses using kmeans (using Pearson correlation, Spearman correlation, and Euclidean distances as distance measures), SC3, cellTree, and Monocle3 (see Supplementary Table 3). In general, we observed strong consistency between many clustering methods with respect to supervised non-spatial and spatial clustering metrics in terms of gene set clustering performance rankings (see Supplementary Fig. 7). Specifically, we observed very similar results in Monocle3 (see Supplementary Fig. 8) to our main results by Leiden clustering, where combining HV and SV genes led to improved clustering performance. For the remainder of the methods, such as SC3, cellTree and kmeans (using pearson correlation, spearman correlation, and Euclidean distance as distance metrics), we also generally observed better results by combining HV and SV genes, as compared to just using either SV genes, HV genes, or both gene sets alone, suggesting a complementary relationship between the two gene sets (see Supplementary Figs. 9–13).

To evaluate the effect of stringency thresholds of HV and SV gene selection, we defined three threshold levels for HV and SV genes: low, medium, and high, based on the gene set and the data platform (see Selection of HV and SV genes). As the threshold level rises, the number of HV and SV genes selected tends to decrease, and the degree of overlap between the respective gene sets also decreases. As shown in Supplementary Fig. 14, as the thresholding level for the HV genes rises, the clustering accuracy tends to decrease, and so does the accuracy of the respective concatenation gene sets. However, we observed an improvement in the clustering accuracy when combining HV and SV genes; nonetheless, regardless of the HV genes’ threshold level. Similarly, as the threshold of SV genes rises, we observe a decrease in clustering accuracy in SV genes and the respective concatenation gene sets. However, combining HV and SV genes improves clustering accuracy regardless (see Supplementary Fig. 15).

### 3.5 Additional robustness checks

To further investigate the robustness of our hypotheses, we also looked into the effect of sequencing depth. Using an example dataset (Visium HER2 Breast Cancer sample B6), we synthetically lowered the sequencing depth of the UMI counts. As shown in Supplementary Fig. 16, clustering performance declined across all gene sets as sequencing depth decreased, yet concatenation consistently matched or outperformed HV genes and SV genes alone across AMI, weighted F1, Pearson Gamma, spatial concordance, and mean spatial AMI. These results indicate that the benefit of concatenation is preserved even under reduced sequencing depth.

We also investigated additional SVG and HVG methods. Using an example dataset (cosMx Non-Small Cell Lung Cancer Patient 3, FOV 24), we applied SpatialDE for SVG selection and LOESS regression for HVG selection. As shown in Supplementary Fig. 17, concatenation provided higher or comparable clustering accuracy across all metrics compared to either method alone. This demonstrates that the advantage of concatenation is robust to the choice of SVG or HVG feature selection approach.

Finally, we include a runtime comparison of the clustering methods as well as PCA, as the number of genes increases. All analyses were performed on a Linux cluster node (Intel(R) Xeon(R) Gold 6140 CPU @ 2.30 GHz, 72 cores, 187 GB RAM). As shown in Supplementary Fig. 18, runtime generally increases with the number of genes. This effect is most pronounced for PCA and for clustering algorithms such as cellTree, which lack an inherent dimension-reduction step. By contrast, clustering methods that incorporate dimension reduction (e.g. Leiden, Monocle3, k-means, and SC3) show comparatively modest fluctuations in runtime. Together, these analyses confirm that despite rare exceptions, the advantage of concatenation is robust across sequencing depth and feature selection methods, and it remains computationally practical for large-scale analyses.

## 4 Discussion

Spatial transcriptomics technologies allow the creation of a more comprehensive map of biological systems. Relative to single cell RNA-Seq technologies, the addition of spatial information has the potential to help discover novel SV markers, which are then used for identifying “spatial domains” in the transcriptomics data; SV genes that share similar spatial expression patterns are also used to define cell types as well as relate cell type composition to tissue structure (Edsgärd *et al.* 2018, Svensson *et al.* 2018, Sun *et al.* 2020, Dries *et al.* 2021, Miller *et al.* 2021). However, these SV genes are not necessarily the best markers to identify biologically insightful clusters, eg, cell types and homogeneity in cell type compositions, a task conventionally accomplished by those HV gene markers (Poirion *et al.* 2016, Zhu *et al.* 2017, Garmire *et al.* 2021, Huang *et al.* 2021). We asked these questions in this study: (1) if SV gene-based clustering can be improved by adding additional HV genes, which are conventionally used in single-cell RNA-Seq and bulk RNA-Seq analysis for clustering; (2) if so, how is the clustering performance of these gene sets affected by clustering method, data platform, and HV and SV selection thresholds? Since spatial transcriptomics platforms are becoming increasingly diverse, it’s important to recognize the platform and tissue context in interpreting cell type or spot-level clustering. We therefore chose to rely on the original study results for ground truth labels. By analyzing multiple datasets across various platforms, we hope to uncover consistent biological truths while minimizing the impact of noise associated with the ground truth.

**Figure 7.**
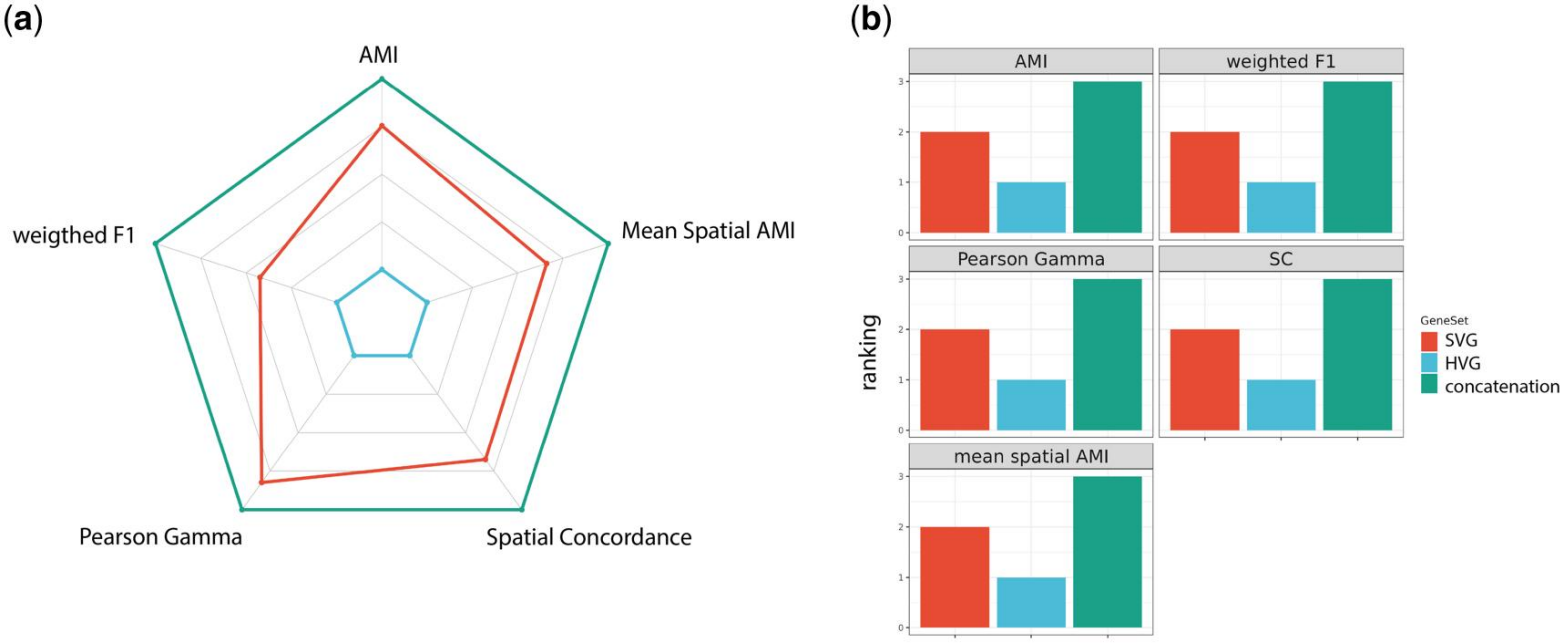
Summary of cell-type clustering performance for HV genes, SV genes, and concatenation. (a) Radar chart using the mean AMI, weighted F1, ASC, Pearson Gamma, Spatial Concordance, and Mean Spatial AMI for HV genes, SV genes, and concatenation. (b) Ranking of HV genes, SV genes, and concatenation for AMI, weighted F1, Pearson Gamma, Spatial Concordance, and Mean Spatial AMI for HV genes, SV genes, and concatenation.

Using multiple metrics, including supervised, unsupervised, and spatially adjusted metrics, as well as closer investigation of the spot/cell level clustering performance, we demonstrated a complementary effect between SV genes and HV genes in terms of cell type clustering. As shown in Fig. 7, clustering metrics show that it is more desirable to use a combination of HV and SV genes rather than either gene s*et al*one. In general, compared to SV genes, HV genes often include more experimentally established markers. In other words, HV genes are conventionally used in scRNA-seq clustering because they are enriched for cell-type-specific marker genes. Thus, adding HV genes likely leads to stronger cell type separation and improved clustering. We also observed platform-specific effects where concatenating HV and SV genes yielded more clustering gains in probe-based platforms (MERFISH, CosMx, and Xenium) than in sequencing-based platforms (Visium). This difference may reflect that probe-based platforms with single-cell resolution are likely to harbor more HVG and SVG due to probe design, making concatenation of the two even more informative. In contrast, Visium’s whole-transcriptome and multi-cell resolution design does not offer the advantages of having enriched HVG/SVGs in the experimental design, leading to smaller improvements when combined. It is also worth noticing that the improvement in concatenation is not simply a result of including more genes. Increasing the gene number does not necessarily improve clustering performance; in fact, adding large numbers of noisy or irrelevant features can dilute meaningful signals, a phenomenon related to the well-known curse of dimensionality (Altman and Krzywinski 2018).

Although rare, there are cases where concatenation did not improve clustering performance compared to HV or SV genes. We examined one such case for the Visium HER2 Breast Cancer Sample E1 (see Supplementary Fig. 19), where AMI scores for SVG, HVG, and concatenation were 0.556, 0.552, and 0.549, respectively. Here, concatenation slightly reduced discrimination between immune-rich cancer cells, cancer 1, and other immune cells. Since the ground-truth cell type labels were manually annotated based on enriched Gene Ontology pathways, it is possible that HVG and SVG did not sufficiently capture the cancer-related or immune function genes used in the original manual cell type annotations. As a result, concatenation did not yield additional clustering benefits in this case.

In summary, since no current gold-standard pipelines exist for obtaining the most biologically insightful clustering in spatial transcriptomics data, our study fills the niche to provide recommendations through conducting a systematic evaluation study. We have confirmed through our evaluation study that combining these two types of markers is a desirable strategy to improve the cell-type clustering accuracy, as compared to the current strategy of using SV genes only for such tasks.

## Supplementary Material

vbaf285_Supplementary_Data

## Data Availability

All data and code used in this evaluation study can be found in the following link https://github.com/lanagarmire/ST_benchmark.
