# Supplementary material for: Adding highly variable genes to spatially variable genes can improve cell type clustering performance in spatial transcriptomics data
Source: Bioinform Adv. 2025 Nov 20;6(1):vbaf285. doi: 10.1093/bioadv/vbaf285 (PMC12809558; doi:10.1093/bioadv/vbaf285)
Supplement: vbaf285_Supplementary_Data [file vbaf285_supplementary_data.zip › Supplementary Materials final.docx]

**Adding Highly Variable Genes to Spatially Var-iable Genes Can Improve Cell Type Clustering Performance in Spatial Transcriptomics Data**

Supplementary Materials

Yijun Li, Stefan Stanojevic, Bing He, Zheng Jing, Qianhui Huang, Jian Kang, Lana X. Garmire

1. **Supplementary Figures**


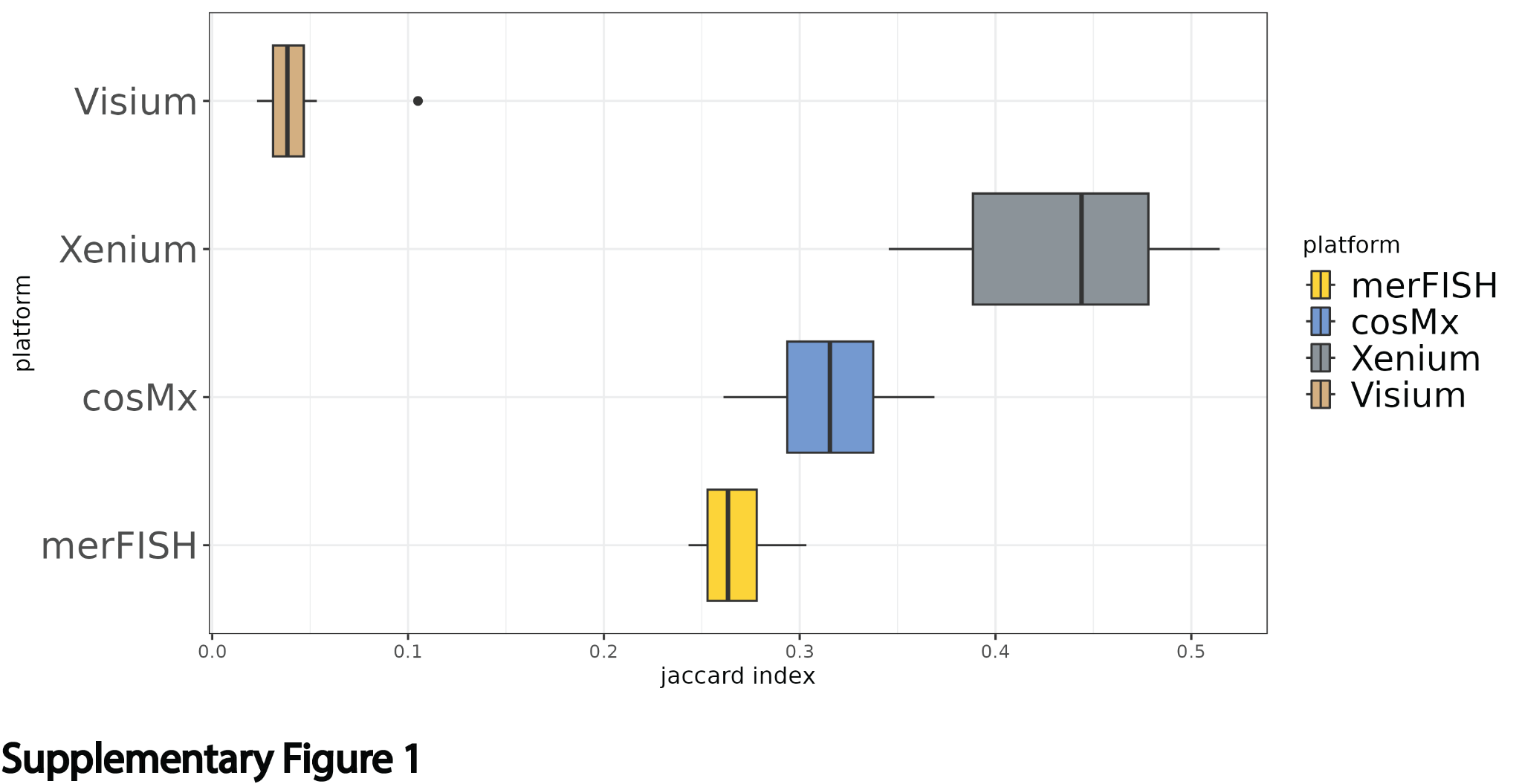


**Supplementary Figure 1.**  Similarity between HVG and SVG for the benchmarked datasets, divided by platform.


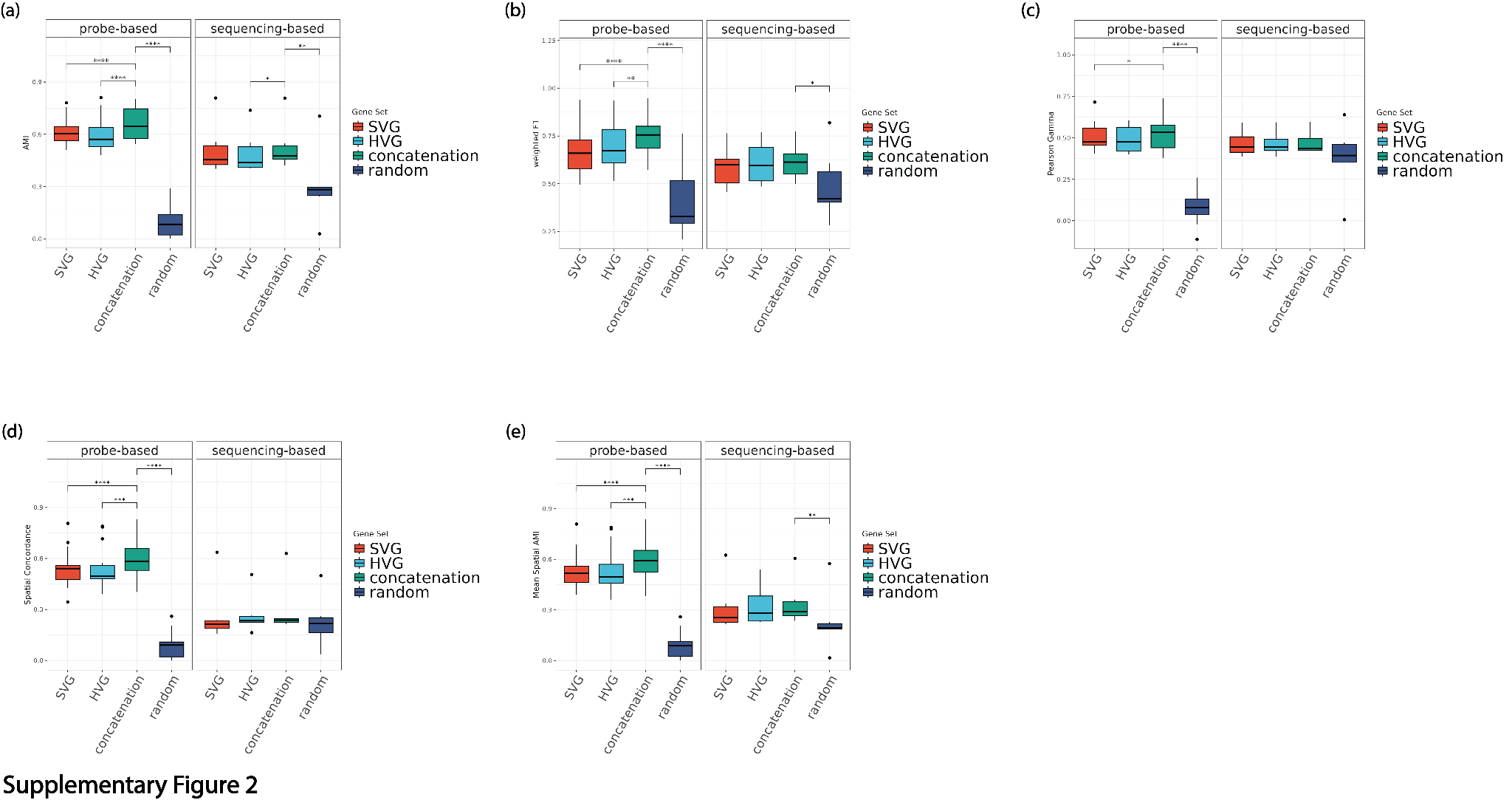


**Supplementary Figure 2**. Comparison of gene set clustering performance of SV genes, HV genes, their union gene set, and average performance of random non-union genes of the same size for Leiden with respect to (a) AMI, (b) weighted F1, (c) Pearson Gamma, (d) Spatial Concordance, (e) Mean Spatial AMI.


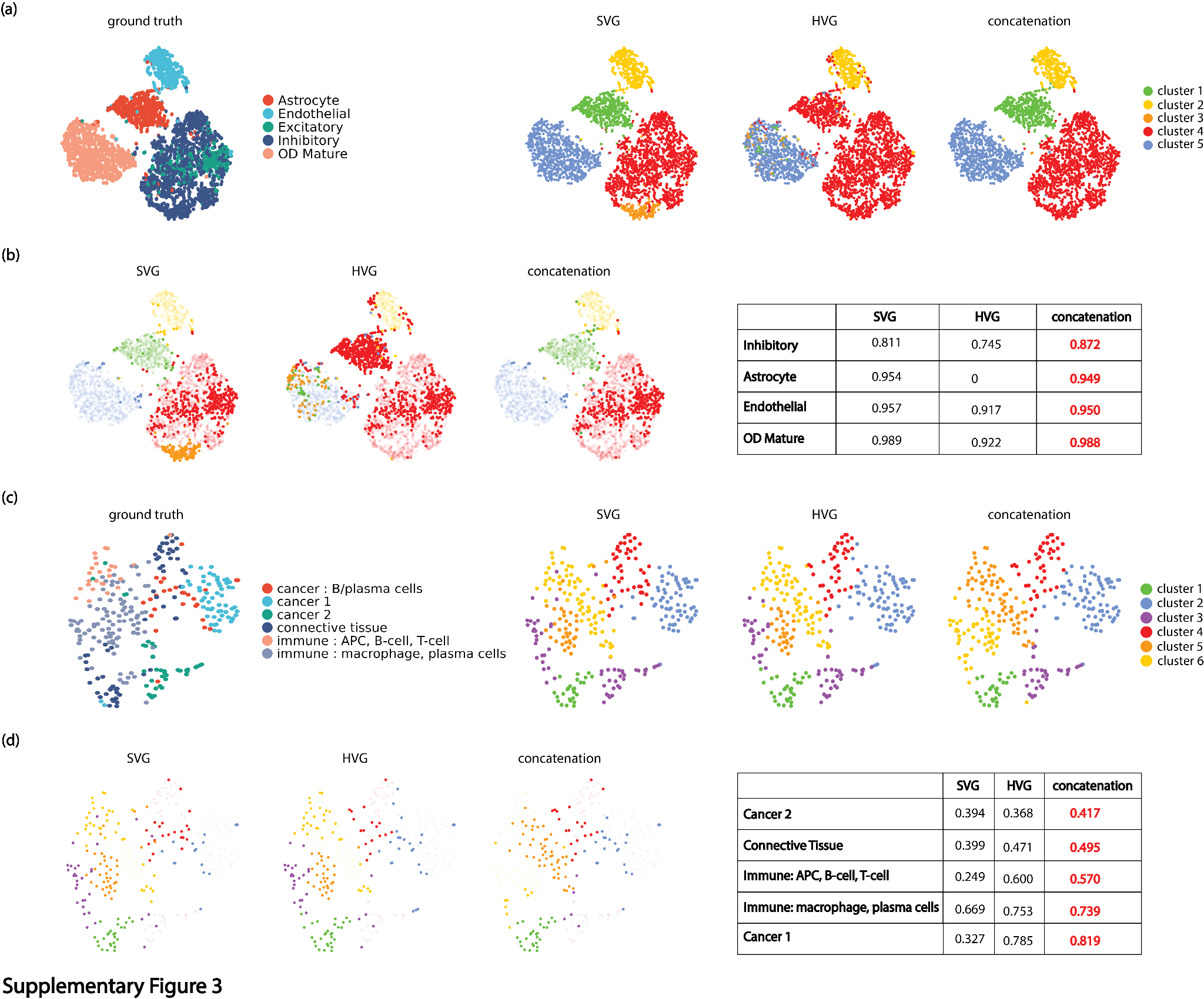


**Supplementary Figure 3.**  Comparison of cluster performance of SV genes, HV genes, and their union set for Leiden for representative datasets on the tSNE space for the union set. (a) comparison of clustering labels for MERFISH mouse hypothalamus dataset (bregma: 210). (b) comparison of tSNE space highlighting mis-classified clusters for each gene set for MERFISH mouse hypothalamus dataset (bregma: 210), with cluster-specific F1 scores for each gene set summarized in a table. (c) comparison of clustering labels for Visium Breast Cancer dataset (sample D1). (d) comparison of tSNE space highlighting mis-classified clusters for each gene set in Visium Breast Cancer dataset (sample D1), with cluster-specific F1 scores for each gene set summarized in a table.


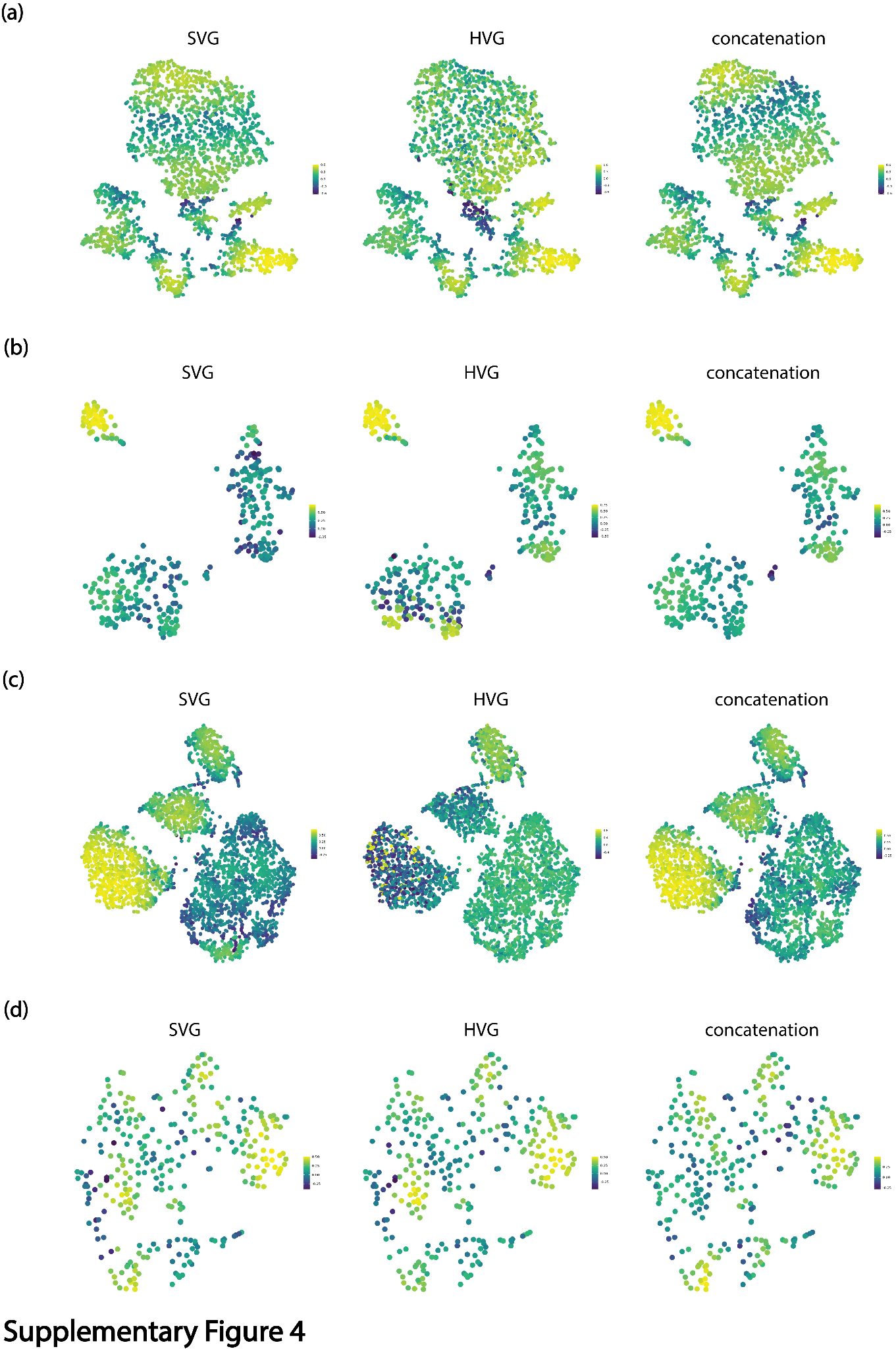


**Supplementary Figure 4**. Comparison of cell / spot-level silhouette widths of representative datasets (a) cosMx NSCLC dataset for patient 2, FOV 7, (b) Xenium Kidney dataset sample N7, (c) MERFISH mouse hypothalamus dataset (bregma: 210), and (d) Visium Breast Cancer dataset, sample D1.


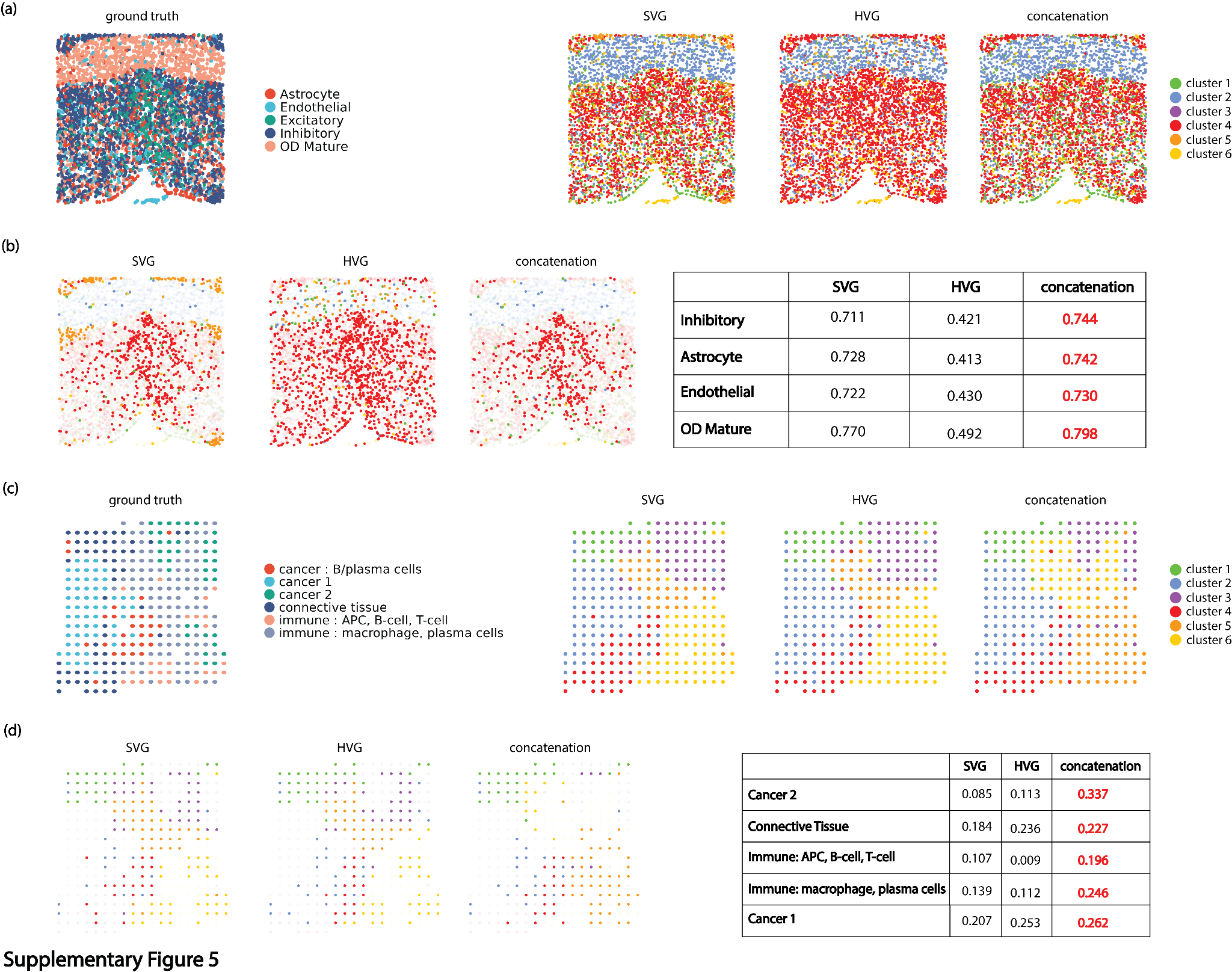


**Supplementary Figure 5.**  Comparison of cluster performance of SV genes, HV genes, and their union set for Leiden for representative datasets on the tissue space. (a) comparison of clustering labels for MERFISH mouse hypothalamus dataset (bregma: 210). (b) comparison of tissue space highlighting mis-classified clusters for each gene set in MERFISH mouse hypothalamus dataset (bregma: 210), with cluster-specific spatial AMI scores for each gene set summarized in a table. (c) comparison of clustering labels for Visium Breast Cancer dataset (sample D1). (d) comparison of tissue space highlighting mis-classified clusters for each gene set in Visium Breast Cancer dataset (sample D1), with cluster-specific spatial AMI scores for each gene set summarized in a table.


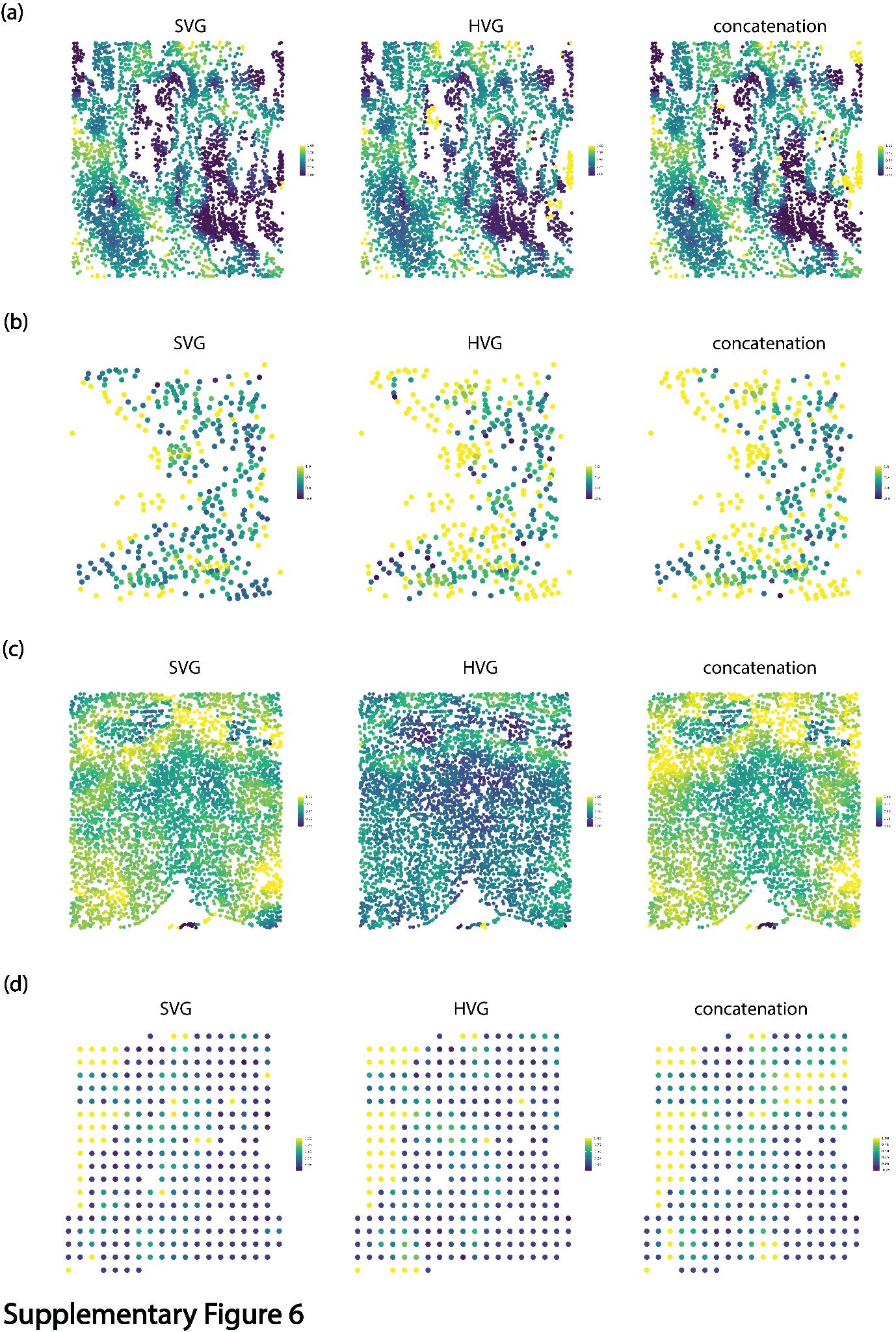


**Supplementary Figure 6**. Comparison of cell / spot-level spatial AMI of representative datasets (a) cosMx NSCLC dataset for patient 2, FOV 7, (b) Xenium Kidney dataset sample N7, (c) MERFISH mouse hypothalamus dataset (bregma: 210), and (d) Visium Breast Cancer dataset, sample D1.


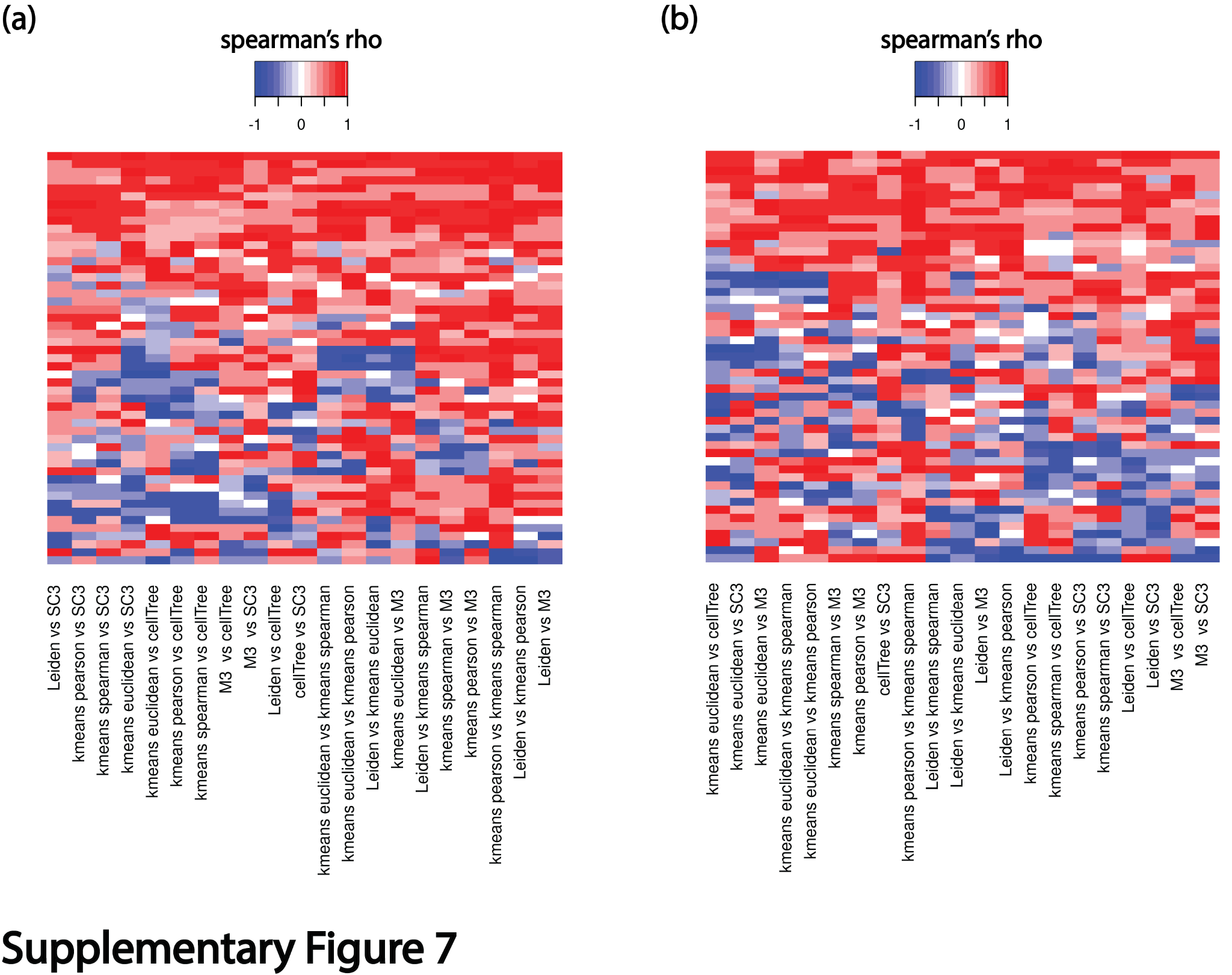


**Supplementary Figure 7.**  (a) Spearman’s rho of the gene sets’ ranking in AMI between different clustering methods. (b) Spearman’s rho of the gene sets’ ranking in Spatial Concordance between different clustering methods.


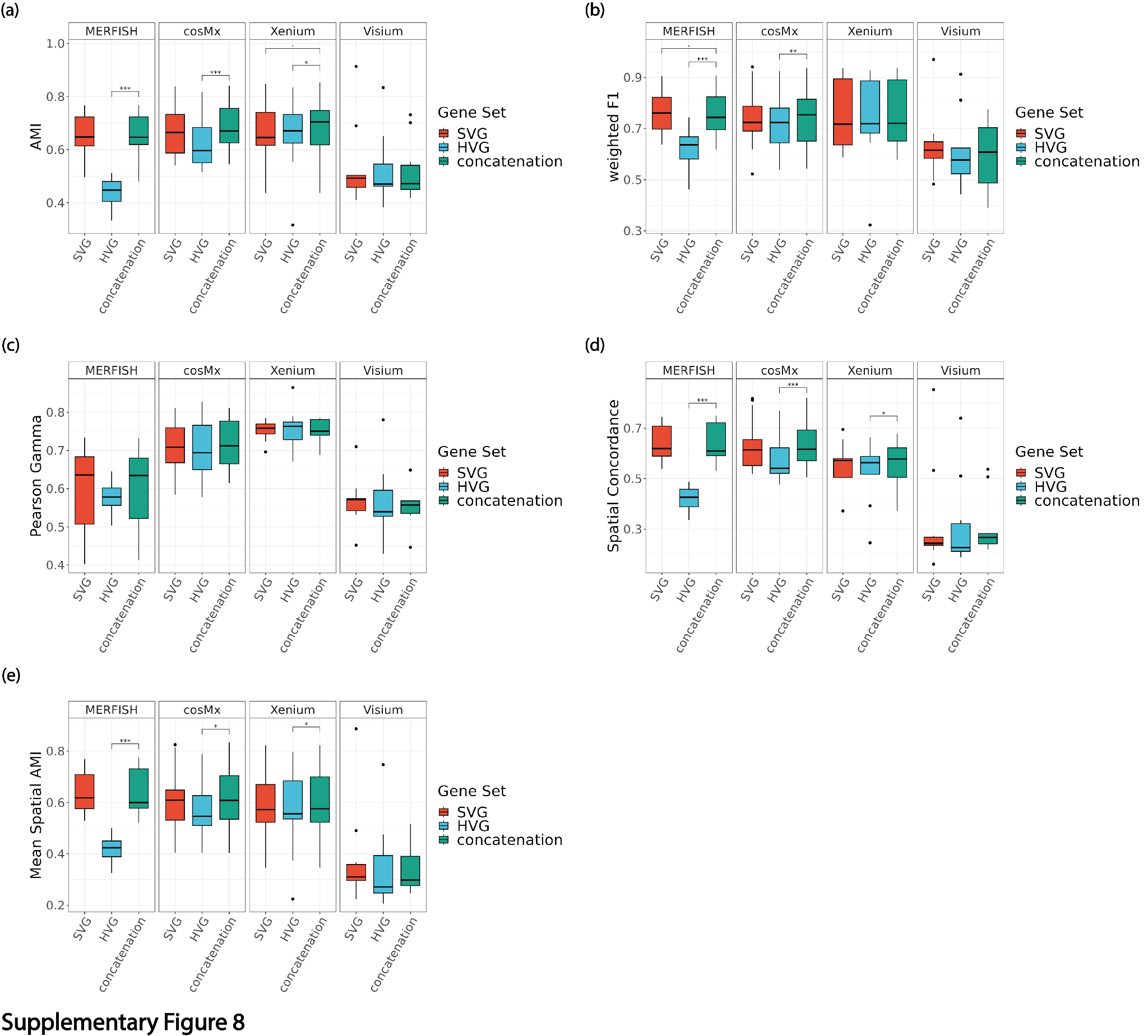


**Supplementary Figure 8.**  Comparison of cluster performance of SV genes, HV genes, their union set, and all genes for Monocle3: (a) AMI. (b) weighted F1. (c) Pearson Gamma. (d) Spatial Concordance. (e) Mean Spatial AMI.

*Note: ***: p-value<1e-3; **: 1e-3* ≤ *p-value* < *1e-2; *: 1e-2* ≤ *p-value* < *5e-2; .: 5e-2* ≤ *p-value < 0.1.*


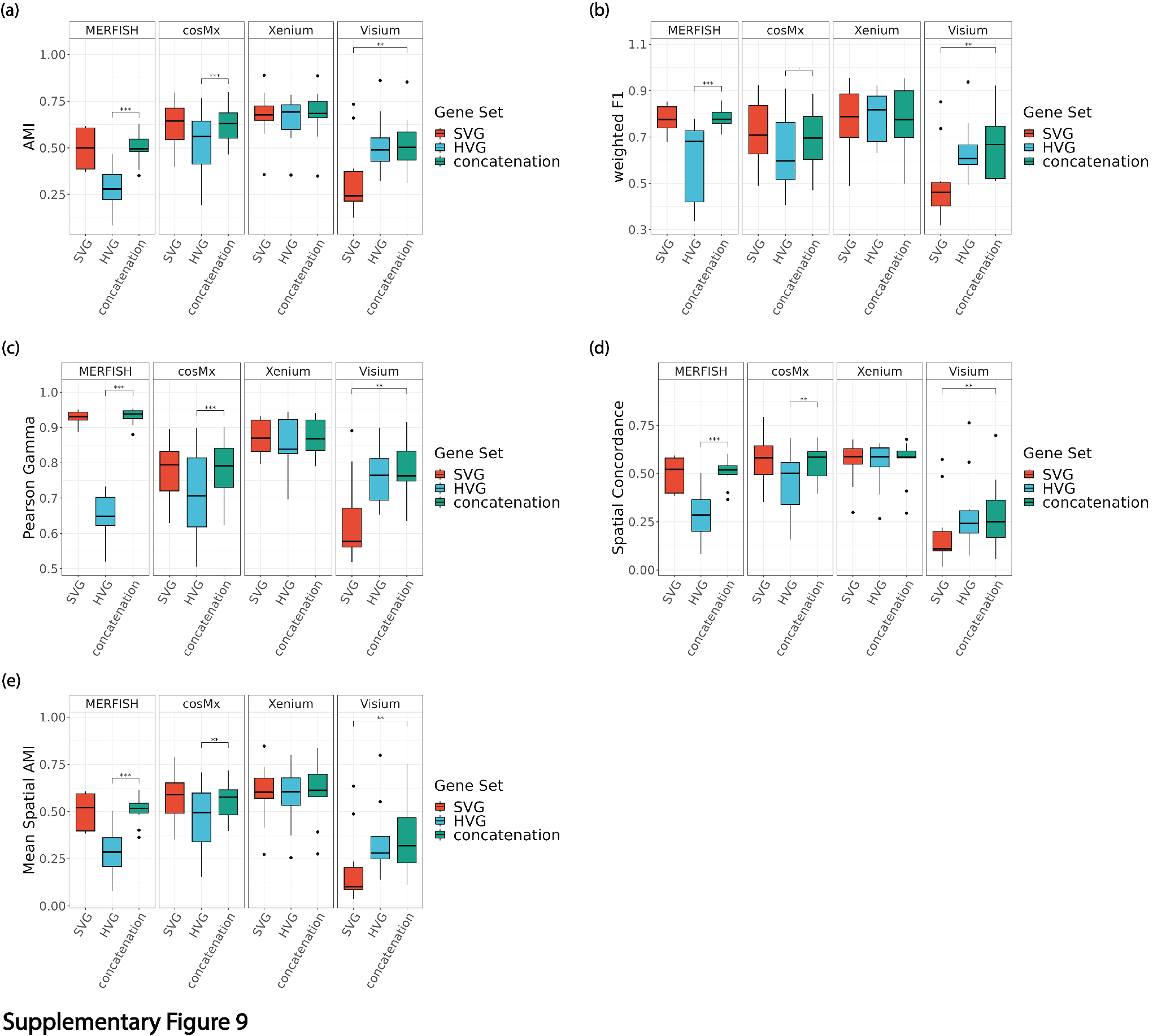


**Supplementary Figure 9.**  Comparison of cluster performance of SV genes, HV genes, their union set, and all genes for SC3: (a) AMI. (b) weighted F1. (c) Pearson Gamma. (d) Spatial Concordance. (e) Mean Spatial AMI.

*Note: ***: p-value<1e-3; **: 1e-3* ≤ *p-value* < *1e-2; *: 1e-2* ≤ *p-value* < *5e-2; .: 5e-2* ≤ *p-value < 0.1.*


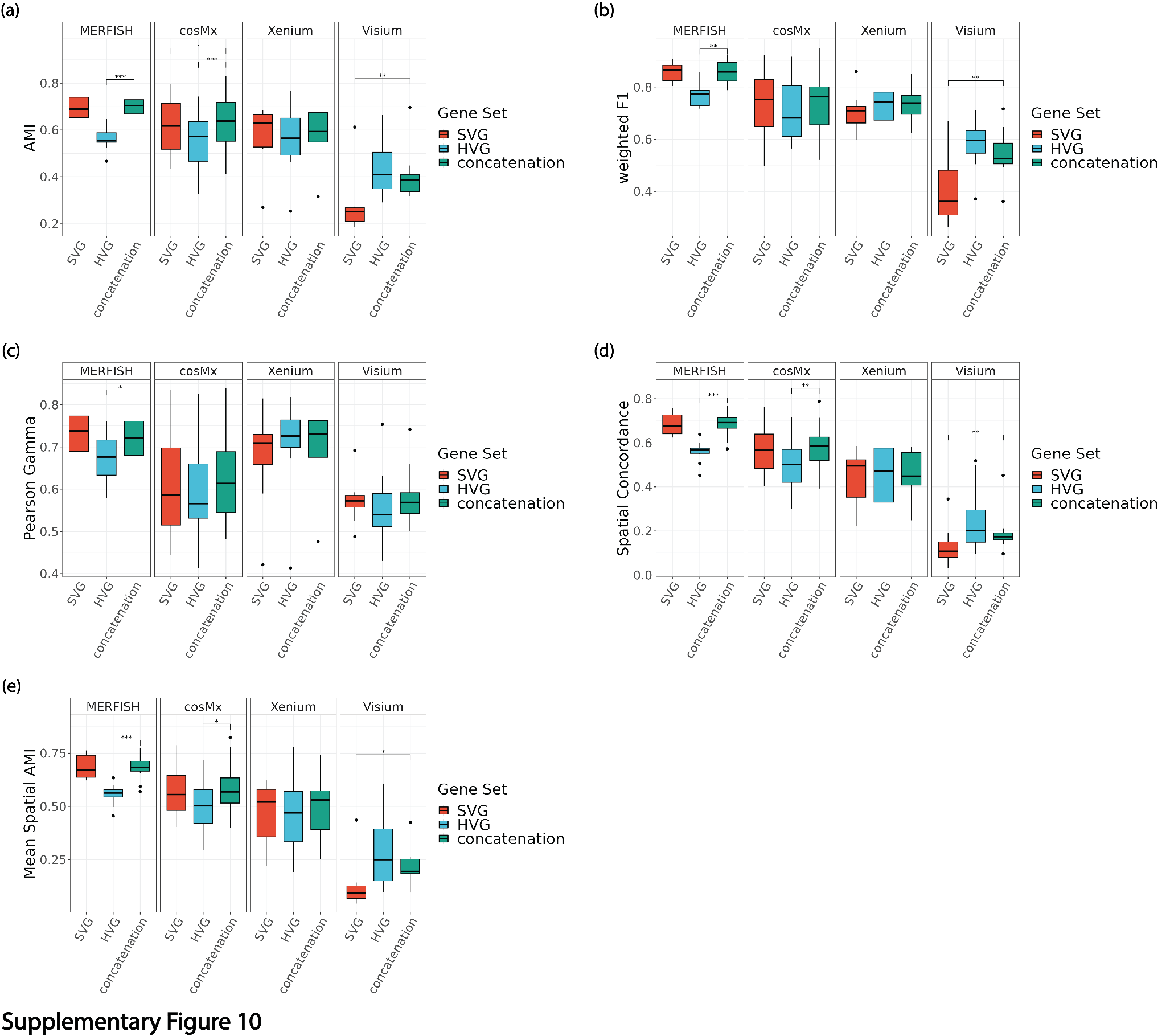


**Supplementary Figure 10.**  Comparison of cluster performance of SV genes, HV genes, their union set, and all genes for cellTree: (a) AMI. (b) weighted F1. (c) Pearson Gamma. (d) Spatial Concordance. (e) Mean Spatial AMI.

*Note: ***: p-value<1e-3; **: 1e-3* ≤ *p-value* < *1e-2; *: 1e-2* ≤ *p-value* < *5e-2; .: 5e-2* ≤ *p-value < 0.1.*


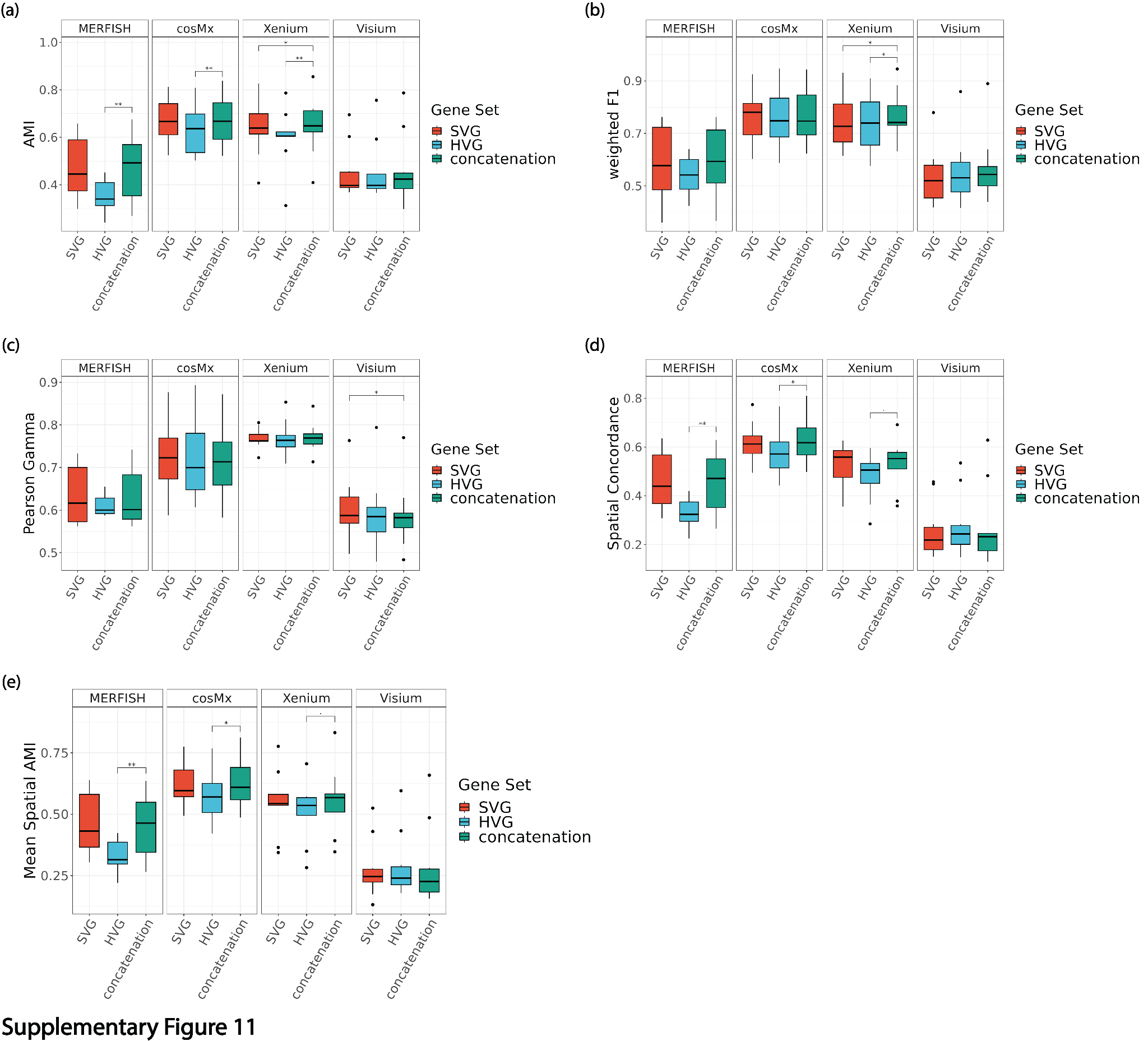


**Supplementary Figure 11.**  Comparison of cluster performance of SV genes, HV genes, their union set, and all genes for kmeans (pearson): (a) AMI. (b) weighted F1. (c) Pearson Gamma. (d) Spatial Concordance. (e) Mean Spatial AMI.

*Note: ***: p-value<1e-3; **: 1e-3* ≤ *p-value* < *1e-2; *: 1e-2* ≤ *p-value* < *5e-2; .: 5e-2* ≤ *p-value < 0.1.*


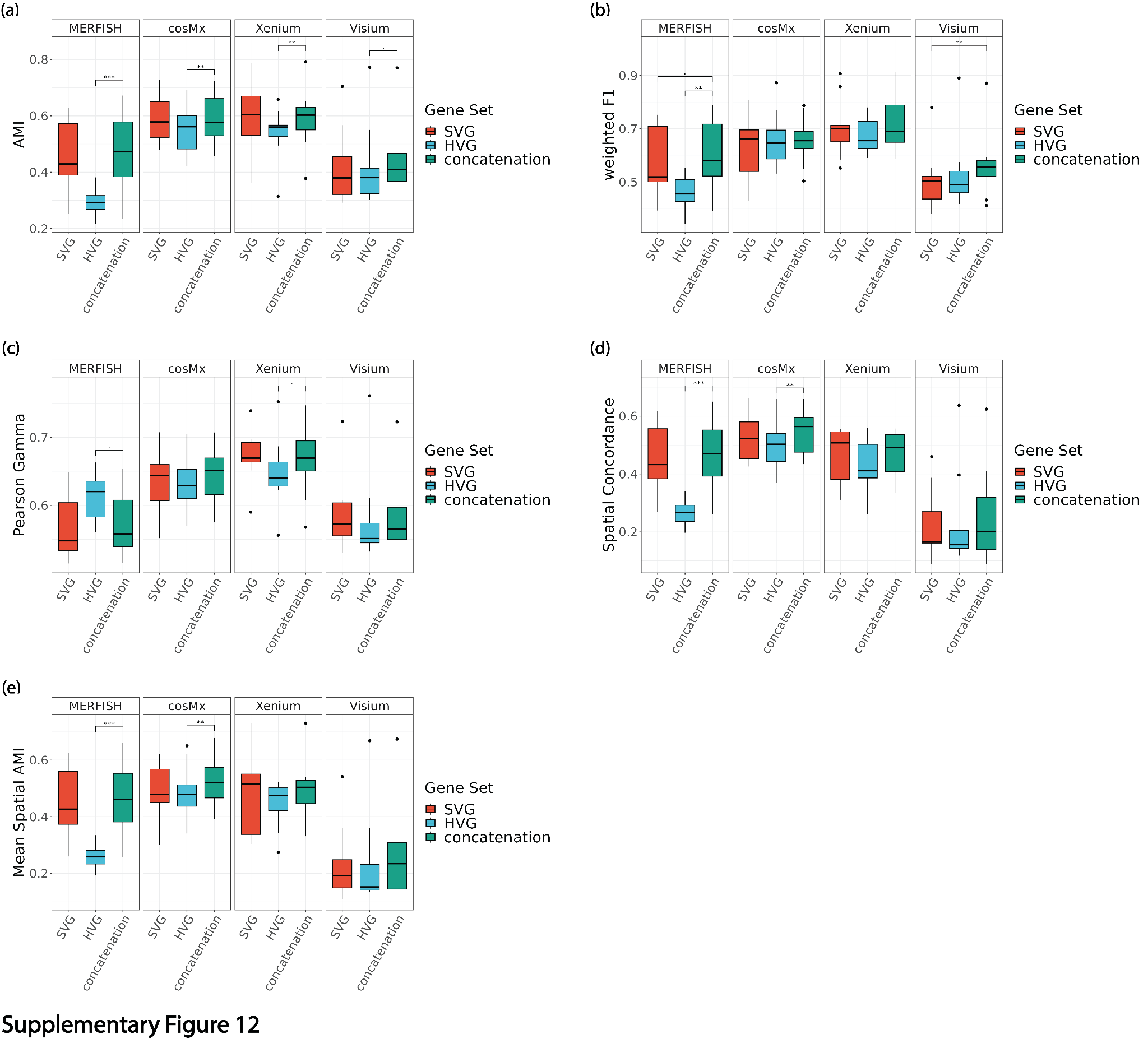


**Supplementary Figure 12.**  Comparison of cluster performance of SV genes, HV genes, their union set, and all genes for kmeans (spearman): (a) AMI. (b) weighted F1. (c) Pearson Gamma. (d) Spatial Concordance. (e) Mean Spatial AMI.

*Note: ***: p-value<1e-3; **: 1e-3* ≤ *p-value* < *1e-2; *: 1e-2* ≤ *p-value* < *5e-2; .: 5e-2* ≤ *p-value < 0.1.*


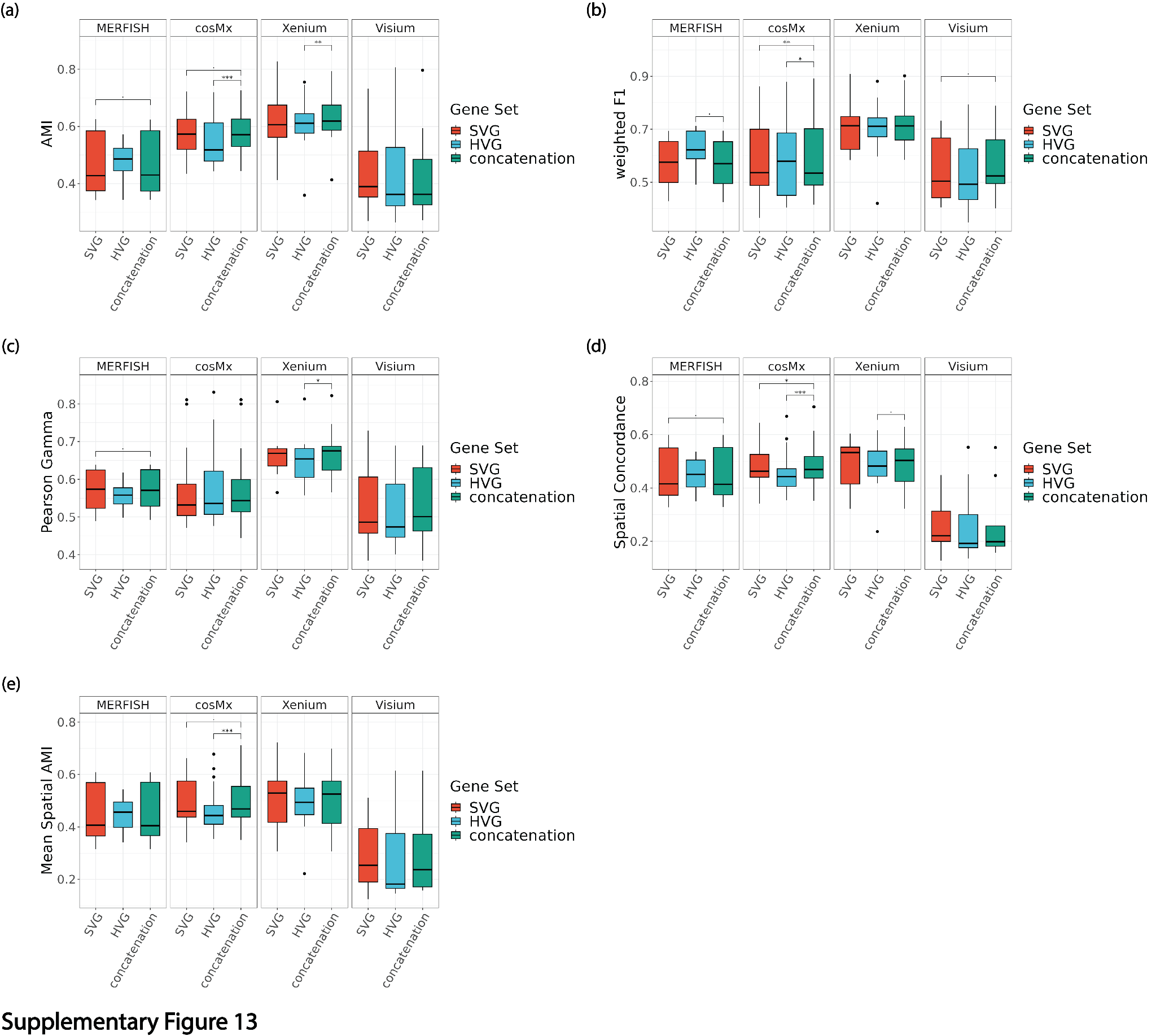


**Supplementary Figure 13.**  Comparison of cluster performance of SV genes, HV genes, their union set, and all genes for kmeans (euclidean): (a) AMI. (b) weighted F1. (c) Pearson Gamma. (d) Spatial Concordance. (e) Mean Spatial AMI.

*Note: ***: p-value<1e-3; **: 1e-3* ≤ *p-value* < *1e-2; *: 1e-2* ≤ *p-value* < *5e-2; .: 5e-2* ≤ *p-value < 0.1.*


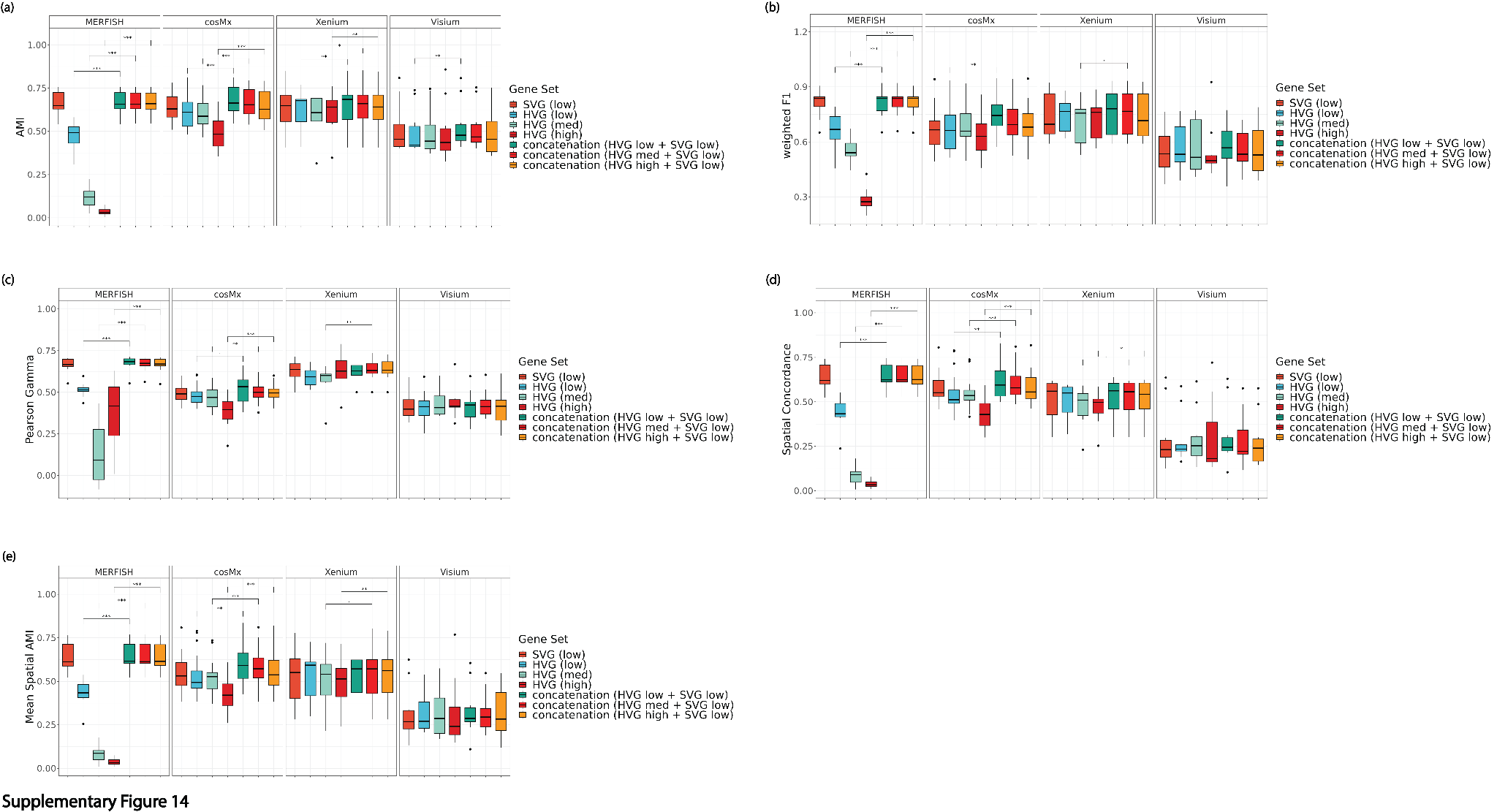


**Supplementary Figure 14.**  Comparisons of clustering performance of real spatial transcriptomics datasets, in four representative platforms including MERFISH, cosMx, Xenium, and Visium at varying HV genes thresholds with respect to (a) AMI, (b) weighted F1, (c) Pearson Gamma, (d) Spatial Concordance, (e) Mean Spatial AMI.

*Note: ***: p-value<1e-3; **: 1e-3* ≤ *p-value* < *1e-2; *: 1e-2* ≤ *p-value* < *5e-2; .: 5e-2* ≤ *p-value < 0.1.*


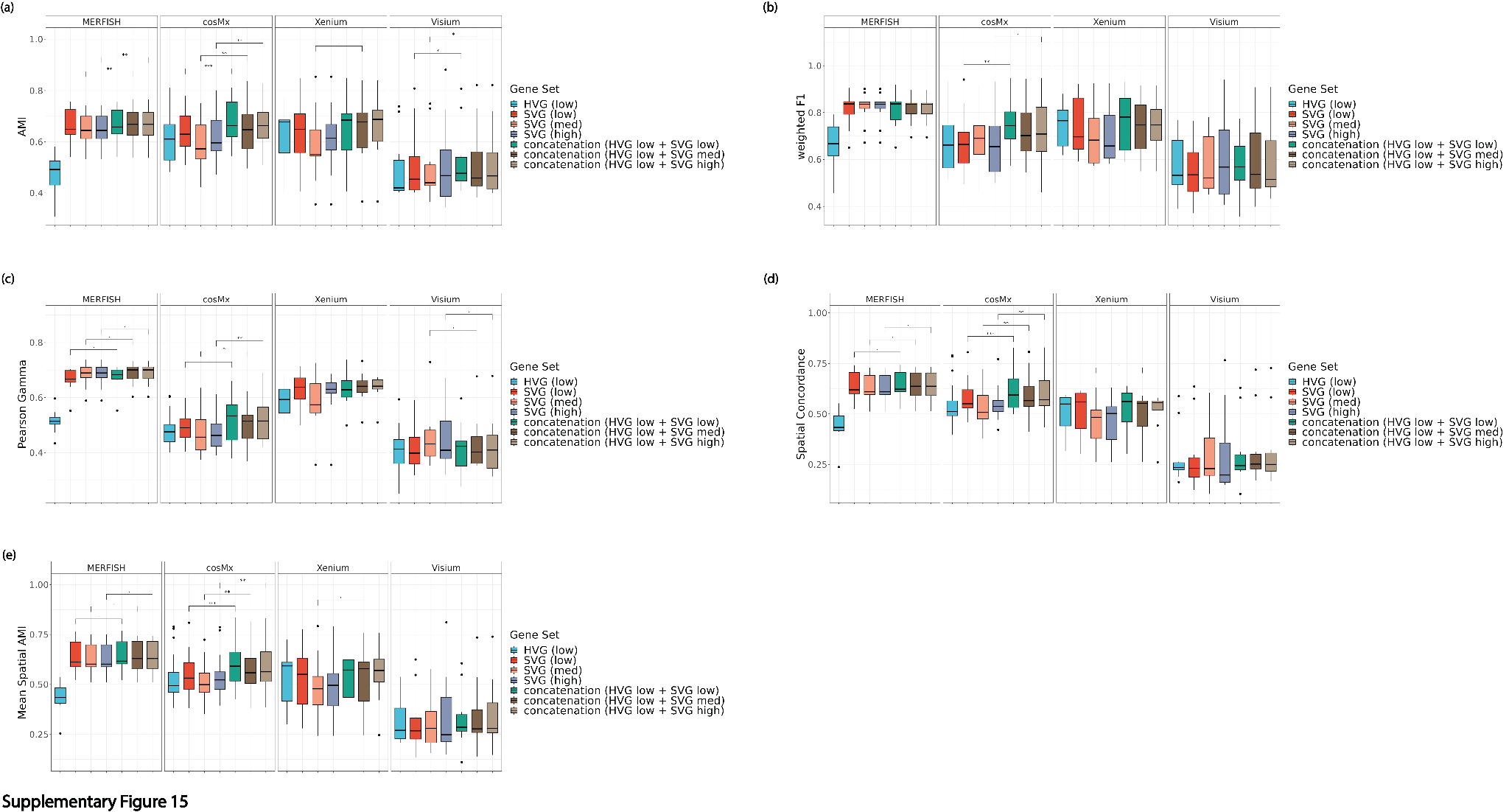


**Supplementary Figure 15.**  Comparisons of clustering performance of real spatial transcriptomics datasets, in four representative platforms including MERFISH, cosMx, Xenium, and Visium at varying SV genes thresholds with respect to (a) AMI, (b) weighted F1, (c) Pearson Gamma, (d) Spatial Concordance, (e) Mean Spatial AMI.

*Note: ***: p-value<1e-3; **: 1e-3* ≤ *p-value* < *1e-2; *: 1e-2* ≤ *p-value* < *5e-2; .: 5e-2* ≤ *p-value < 0.1.*

*
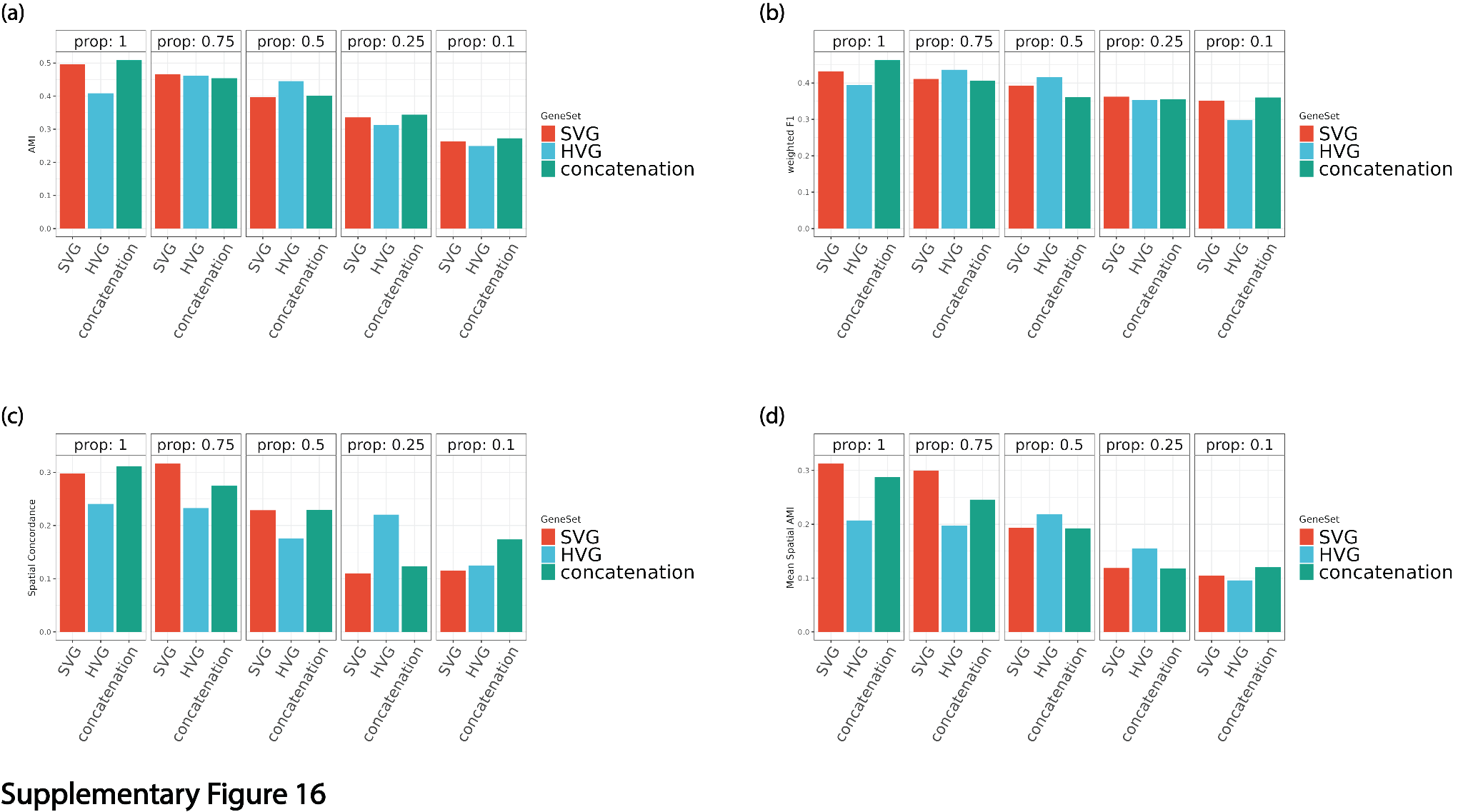
*

**Supplementary Figure 16.**  Clustering performance of HV genes, SV genes, and concatenation of a representative dataset HER2 Breast Cancer sample B6 with varying degrees of sequencing depths with respect to (a) AMI, (b) weighted F1, (c) Spatial Concordance, (d) Mean Spatial AMI.

**
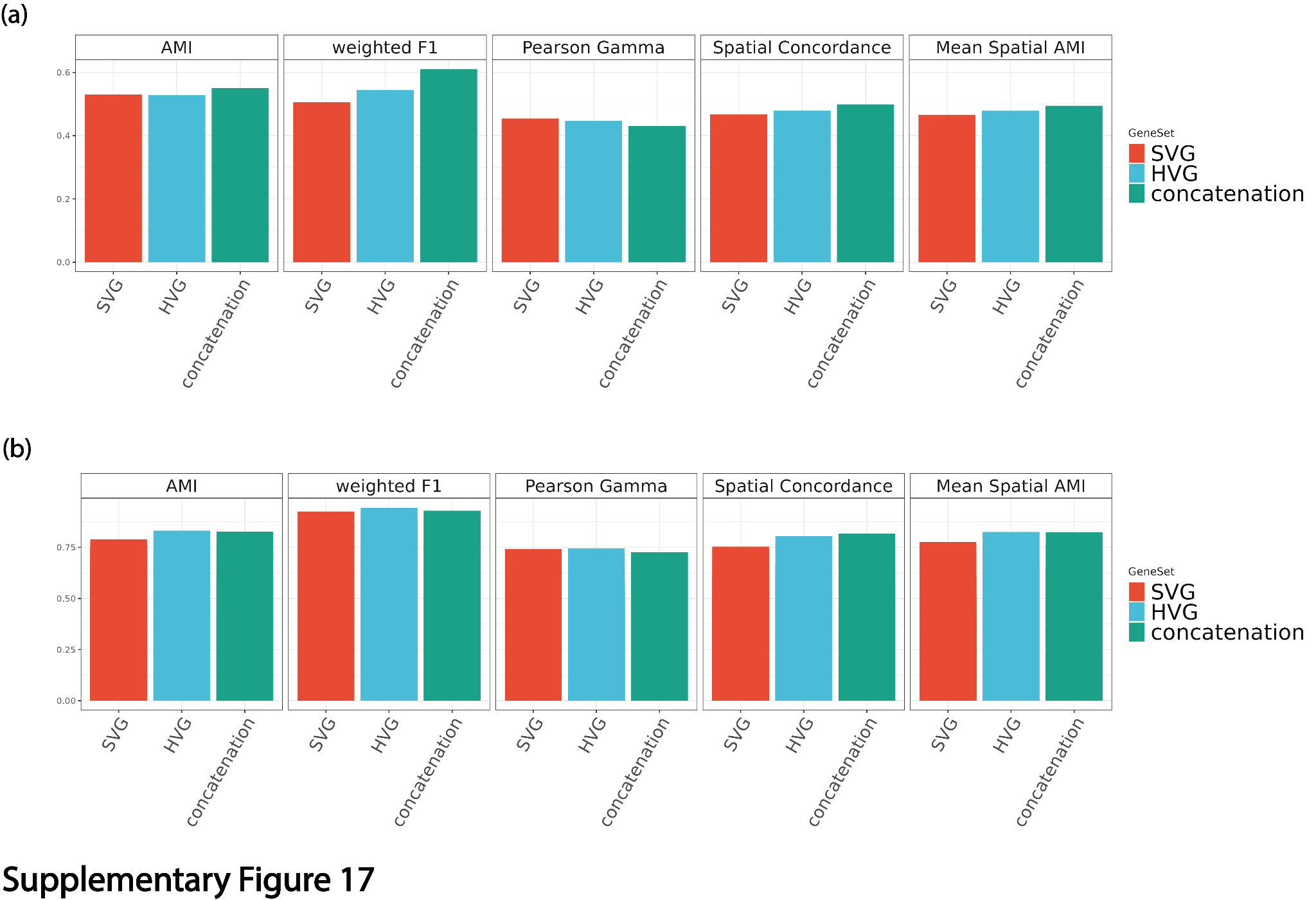
**

**Supplementary Figure 17.**  Comparison of cluster performance of SV genes, HV genes, and their union set for Leiden for cosMx Non-Small Cell Lung Cancer Patient 3, FOV 24. (a) AMI, weighted F1, Pearson Gamma, Spatial Concordance, and Mean Spatial AMI for SV genes obtained with SPARK, HV genes obtained with SCT, and their union set. (b) AMI, weighted F1, Pearson Gamma, Spatial Concordance, and Mean Spatial AMI for SV genes obtained with spatialDE, HV genes obtained with LOESS smoothing, and their union set. (c) Venn diagram of HV genes obtained with VST and LOESS. (d) Venn diagram of SV genes obtained with SPARK and spatialDE.


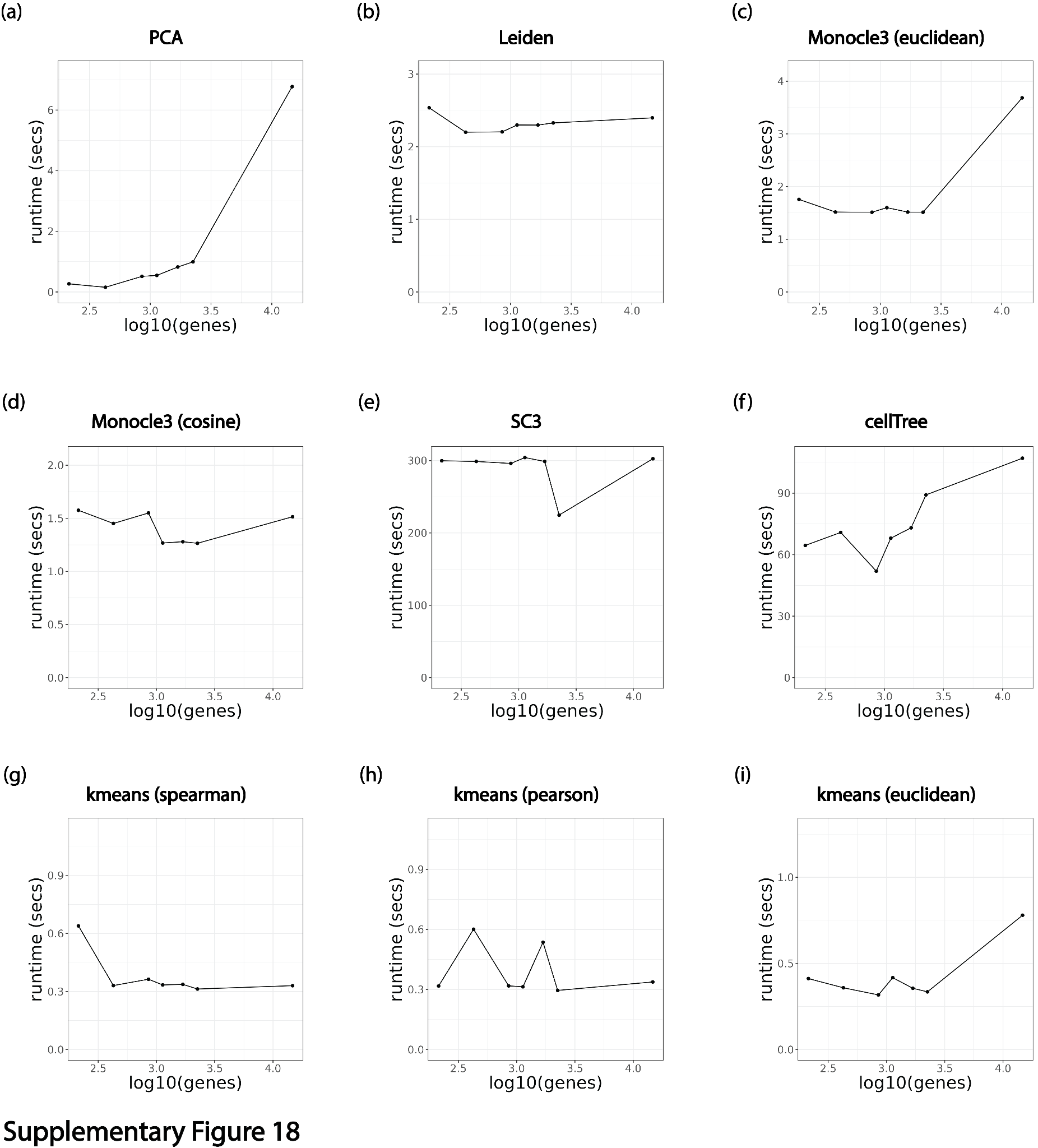


**Supplementary Figure 18**. Runtime analysis as the number of genes increase for (a) PCA, (b) Leiden, (c) Monocle3 (euclidean), (d) Monocle3 (cosine), (e) SC3, (f) cellTree, (g) kmeans (spearman), (h) kmeans (pearson), (i) kmeans (euclidean). All analyses were run on a Linux cluster node (Intel(R) Xeon(R) Gold 6140 CPU @ 2.30GHz, 72 cores, 187 GB RAM.


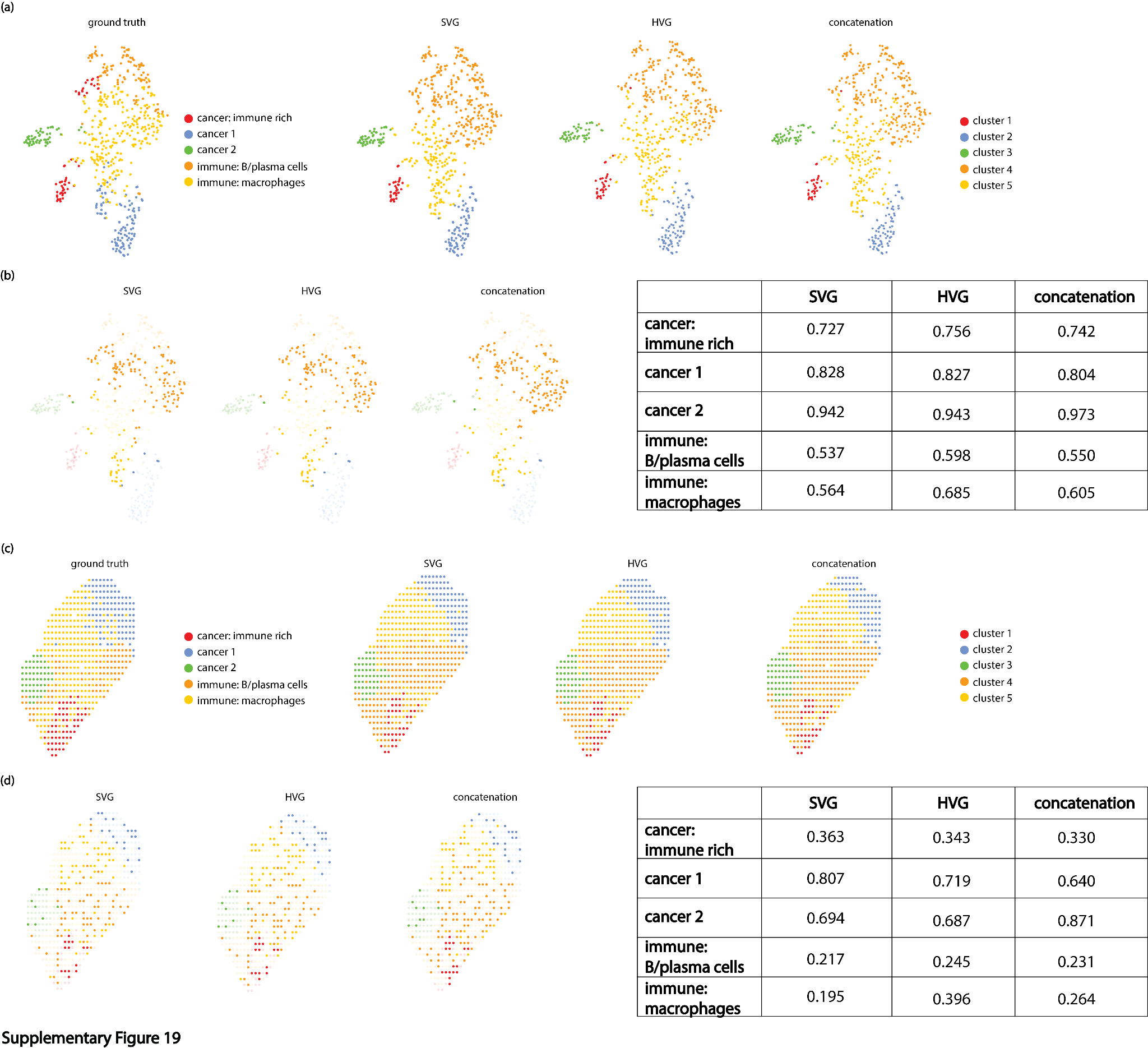


**Supplementary Figure 19.**  Comparison of cluster performance of SV genes, HV genes, and their union set for Leiden for HER2 Breast Cancer Sample E1. (a) comparison of clustering labels in the tSNE space. (b) comparison of tSNE space highlighting mis-classified clusters for each gene set, with cluster-specific spatial AMI scores for each gene set summarized in a table. (c) comparison of clustering labels in the tissue space. (d) comparison of tissue space highlighting mis-classified clusters for each gene set, with cluster-specific spatial AMI scores for each gene set summarized in a table.

1. **Supplementary Tables**

**Supplementary Table 1**: Summary of highly variable genes and spatially variable genes at different threshold levels.

| Dataset | HV genes (low) | HV genes (med) | HV genes (high) | SV genes (low) | SV genes (med) | SV genes (high) |
| --- | --- | --- | --- | --- | --- | --- |
| Mouse Brain Olfactory Bulb Dataset (replicate 1) | 2339 | 1404 | 468 | 973 | 487 | 244 |
| HER2 Breast Cancer Dataset (sample A3) | 1833 | 1100 | 367 | 720 | 360 | 180 |
| HER2 Breast Cancer Dataset (sample B3) | 1255 | 753 | 251 | 713 | 357 | 179 |
| HER2 Breast Cancer Dataset (sample B6) | 1502 | 901 | 301 | 854 | 427 | 214 |
| HER2 Breast Cancer Dataset (sample D1) | 2175 | 1305 | 435 | 595 | 298 | 149 |
| HER2 Breast Cancer Dataset (sample D2) | 1951 | 1171 | 391 | 733 | 367 | 184 |
| HER2 Breast Cancer Dataset (sample D3) | 1953 | 1172 | 391 | 719 | 360 | 180 |
| HER2 Breast Cancer Dataset (sample D5) | 1865 | 1119 | 373 | 607 | 304 | 152 |
| HER2 Breast Cancer Dataset (sample E1) | 951 | 571 | 191 | 528 | 264 | 132 |
| Ovarian Cancer Dataset (sample B_GTFB1191) | 1742 | 1045 | 349 | 10101 | 5051 | 3684 |
| Human Kidney Dataset (sample N7) | 87 | 68 | 49 | 106 | 53 | 27 |
| Human Kidney Dataset (sample O7) | 100 | 78 | 56 | 162 | 81 | 50 |
| Human Kidney Dataset (sample O8) | 124 | 96 | 69 | 205 | 108 | 78 |
| Human Kidney Dataset (sample R7) | 100 | 78 | 56 | 222 | 111 | 111 |
| Human Kidney Dataset (sample S7) | 113 | 88 | 63 | 224 | 128 | 108 |
| Human Kidney Dataset (sample T7) | 110 | 86 | 62 | 211 | 106 | 69 |
| Human Kidney Dataset (sample U7) | 101 | 79 | 57 | 198 | 99 | 65 |
| Mouse Brain ATN Dataset (sample S1 anterior) | 79 | 61 | 44 | 178 | 92 | 82 |
| Mouse Brain ATN Dataset (sample S2 interior) | 83 | 65 | 47 | 189 | 128 | 128 |
| Mouse Brain Hypothalamus Dataset (bregma: -140) | 42 | 25 | 9 | 147 | 107 | 107 |
| Mouse Brain Hypothalamus Dataset (bregma: -190) | 42 | 25 | 9 | 148 | 105 | 105 |
| Mouse Brain Hypothalamus Dataset (bregma: -240) | 43 | 26 | 9 | 139 | 99 | 99 |
| Mouse Brain Hypothalamus Dataset (bregma: -290) | 43 | 26 | 9 | 141 | 102 | 102 |
| Mouse Brain Hypothalamus Dataset (bregma: -40) | 43 | 26 | 9 | 147 | 101 | 101 |
| Mouse Brain Hypothalamus Dataset (bregma: -90) | 42 | 25 | 9 | 146 | 106 | 106 |
| Mouse Brain Hypothalamus Dataset (bregma: 10) | 44 | 26 | 9 | 144 | 114 | 114 |
| Mouse Brain Hypothalamus Dataset (bregma: 110) | 39 | 24 | 8 | 144 | 98 | 98 |
| Mouse Brain Hypothalamus Dataset (bregma: 160) | 46 | 28 | 10 | 143 | 101 | 101 |
| Mouse Brain Hypothalamus Dataset (bregma: 210) | 43 | 26 | 9 | 141 | 98 | 98 |
| Mouse Brain Hypothalamus Dataset (bregma: 260) | 40 | 24 | 8 | 139 | 82 | 82 |
| Mouse Brain Hypothalamus Dataset (bregma: 60) | 43 | 26 | 9 | 148 | 107 | 107 |
| Human Lung NSCLC Dataset (patient 5, Rep 1, FOV 1) | 154 | 92 | 31 | 300 | 150 | 86 |
| Human Lung NSCLC Dataset (patient 5, Rep 1, FOV 23) | 191 | 115 | 39 | 307 | 154 | 82 |
| Human Lung NSCLC Dataset (patient 5, Rep 1, FOV 25) | 157 | 94 | 32 | 360 | 180 | 110 |
| Human Lung NSCLC Dataset (patient 5, Rep 2, FOV 14) | 279 | 168 | 56 | 204 | 102 | 56 |
| Human Lung NSCLC Dataset (patient 5, Rep 2, FOV 18) | 255 | 153 | 51 | 357 | 179 | 107 |
| Human Lung NSCLC Dataset (patient 5, Rep 2, FOV 20) | 184 | 111 | 37 | 407 | 204 | 133 |
| Human Lung NSCLC Dataset (patient 5, Rep 2, FOV 21) | 223 | 134 | 45 | 330 | 165 | 103 |
| Human Lung NSCLC Dataset (patient 5, Rep 2, FOV 24) | 217 | 130 | 44 | 208 | 104 | 61 |
| Human Lung NSCLC Dataset (patient 5, Rep 2, FOV 7) | 215 | 129 | 43 | 581 | 339 | 289 |
| Human Lung NSCLC Dataset (patient 5, Rep 3, FOV 12) | 142 | 85 | 29 | 492 | 259 | 204 |
| Human Lung NSCLC Dataset (patient 5, Rep 3, FOV 13) | 156 | 94 | 32 | 422 | 211 | 143 |
| Human Lung NSCLC Dataset (patient 5, Rep 3, FOV 14) | 173 | 104 | 35 | 228 | 114 | 59 |
| Human Lung NSCLC Dataset (patient 5, Rep 3, FOV 15) | 184 | 110 | 37 | 503 | 260 | 210 |
| Human Lung NSCLC Dataset (patient 5, Rep 3, FOV 19) | 162 | 97 | 33 | 331 | 166 | 83 |
| Human Lung NSCLC Dataset (patient 5, Rep 3, FOV 22) | 180 | 108 | 36 | 328 | 164 | 90 |
| Human Lung NSCLC Dataset (patient 5, Rep 3, FOV 24) | 151 | 91 | 31 | 247 | 124 | 74 |
| Human Lung NSCLC Dataset (patient 5, Rep 3, FOV 25) | 132 | 79 | 27 | 303 | 152 | 78 |
| Human Lung NSCLC Dataset (patient 5, Rep 3, FOV 27) | 143 | 86 | 29 | 417 | 213 | 162 |
| Human Lung NSCLC Dataset (patient 5, Rep 3, FOV 28) | 204 | 123 | 41 | 605 | 322 | 283 |
| Human Lung NSCLC Dataset (patient 5, Rep 3, FOV 30) | 144 | 87 | 29 | 345 | 173 | 93 |

**Supplementary Table 2**: Summary of concatenation gene sets at different highly variable genes and spatially variable genes threshold levels.

| Dataset | Concatenation (HV genes low + SV genes low) | Concatenation (HV genes low + SV genes med) | Concatenation (HV genes low + SV genes high) | Concatenation (HV genes med + SV genes low) | Concatenation (HV genes high + SV genes low) |
| --- | --- | --- | --- | --- | --- |
| Mouse Brain Olfactory Bulb Dataset (replicate 1) | 3144 | 2738 | 2539 | 2271 | 1406 |
| HER2 Breast Cancer Dataset (sample A3) | 2488 | 2139 | 1994 | 1753 | 1067 |
| HER2 Breast Cancer Dataset (sample B3) | 1913 | 1587 | 1418 | 1430 | 957 |
| HER2 Breast Cancer Dataset (sample B6) | 2248 | 1876 | 1684 | 1690 | 1132 |
| HER2 Breast Cancer Dataset (sample D1) | 2689 | 2430 | 2305 | 1849 | 1010 |
| HER2 Breast Cancer Dataset (sample D2) | 2590 | 2267 | 2107 | 1846 | 1108 |
| HER2 Breast Cancer Dataset (sample D3) | 2568 | 2247 | 2102 | 1832 | 1092 |
| HER2 Breast Cancer Dataset (sample D5) | 2391 | 2128 | 1999 | 1680 | 965 |
| HER2 Breast Cancer Dataset (sample E1) | 1446 | 1199 | 1076 | 1077 | 708 |
| Ovarian Cancer Dataset (sample B_GTFB1191) | 10717 | 6246 | 5024 | 10476 | 10223 |
| Human Kidney Dataset (sample N7) | 139 | 109 | 97 | 128 | 120 |
| Human Kidney Dataset (sample O7) | 173 | 119 | 107 | 167 | 164 |
| Human Kidney Dataset (sample O8) | 224 | 153 | 142 | 218 | 214 |
| Human Kidney Dataset (sample R7) | 223 | 134 | 134 | 223 | 222 |
| Human Kidney Dataset (sample S7) | 228 | 160 | 145 | 227 | 225 |
| Human Kidney Dataset (sample T7) | 216 | 144 | 128 | 216 | 214 |
| Human Kidney Dataset (sample U7) | 210 | 134 | 114 | 206 | 203 |
| Mouse Brain ATN Dataset (sample S1 anterior) | 191 | 122 | 113 | 183 | 180 |
| Mouse Brain ATN Dataset (sample S2 interior) | 200 | 153 | 153 | 197 | 193 |
| Mouse Brain Hypothalamus Dataset (bregma: -140) | 149 | 125 | 125 | 148 | 148 |
| Mouse Brain Hypothalamus Dataset (bregma: -190) | 149 | 120 | 120 | 149 | 149 |
| Mouse Brain Hypothalamus Dataset (bregma: -240) | 146 | 117 | 117 | 146 | 142 |
| Mouse Brain Hypothalamus Dataset (bregma: -290) | 148 | 119 | 119 | 147 | 144 |
| Mouse Brain Hypothalamus Dataset (bregma: -40) | 151 | 115 | 115 | 151 | 149 |
| Mouse Brain Hypothalamus Dataset (bregma: -90) | 150 | 121 | 121 | 149 | 148 |
| Mouse Brain Hypothalamus Dataset (bregma: 10) | 148 | 125 | 125 | 148 | 148 |
| Mouse Brain Hypothalamus Dataset (bregma: 110) | 146 | 114 | 114 | 145 | 145 |
| Mouse Brain Hypothalamus Dataset (bregma: 160) | 145 | 118 | 118 | 145 | 143 |
| Mouse Brain Hypothalamus Dataset (bregma: 210) | 143 | 115 | 115 | 143 | 141 |
| Mouse Brain Hypothalamus Dataset (bregma: 260) | 143 | 104 | 104 | 143 | 141 |
| Mouse Brain Hypothalamus Dataset (bregma: 60) | 148 | 123 | 123 | 148 | 148 |
| Human Lung NSCLC Dataset (patient 5, Rep 1, FOV 1) | 357 | 239 | 207 | 323 | 300 |
| Human Lung NSCLC Dataset (patient 5, Rep 1, FOV 23) | 367 | 261 | 216 | 327 | 311 |
| Human Lung NSCLC Dataset (patient 5, Rep 1, FOV 25) | 398 | 256 | 206 | 375 | 361 |
| Human Lung NSCLC Dataset (patient 5, Rep 2, FOV 14) | 383 | 320 | 296 | 290 | 213 |
| Human Lung NSCLC Dataset (patient 5, Rep 2, FOV 18) | 456 | 332 | 304 | 396 | 363 |
| Human Lung NSCLC Dataset (patient 5, Rep 2, FOV 20) | 452 | 287 | 250 | 419 | 407 |
| Human Lung NSCLC Dataset (patient 5, Rep 2, FOV 21) | 404 | 292 | 262 | 355 | 330 |
| Human Lung NSCLC Dataset (patient 5, Rep 2, FOV 24) | 328 | 265 | 241 | 268 | 214 |
| Human Lung NSCLC Dataset (patient 5, Rep 2, FOV 7) | 590 | 381 | 352 | 583 | 581 |
| Human Lung NSCLC Dataset (patient 5, Rep 3, FOV 12) | 502 | 295 | 256 | 494 | 492 |
| Human Lung NSCLC Dataset (patient 5, Rep 3, FOV 13) | 449 | 285 | 237 | 432 | 422 |
| Human Lung NSCLC Dataset (patient 5, Rep 3, FOV 14) | 305 | 223 | 194 | 257 | 233 |
| Human Lung NSCLC Dataset (patient 5, Rep 3, FOV 15) | 520 | 341 | 309 | 507 | 503 |
| Human Lung NSCLC Dataset (patient 5, Rep 3, FOV 19) | 369 | 240 | 190 | 344 | 332 |
| Human Lung NSCLC Dataset (patient 5, Rep 3, FOV 22) | 386 | 267 | 226 | 350 | 330 |
| Human Lung NSCLC Dataset (patient 5, Rep 3, FOV 24) | 294 | 206 | 174 | 258 | 248 |
| Human Lung NSCLC Dataset (patient 5, Rep 3, FOV 25) | 329 | 215 | 163 | 313 | 305 |
| Human Lung NSCLC Dataset (patient 5, Rep 3, FOV 27) | 435 | 269 | 237 | 421 | 418 |
| Human Lung NSCLC Dataset (patient 5, Rep 3, FOV 28) | 618 | 383 | 358 | 608 | 605 |
| Human Lung NSCLC Dataset (patient 5, Rep 3, FOV 30) | 371 | 231 | 187 | 355 | 346 |

**Supplementary Table 3**: Summary of clustering methods

| **Method** | **Required input data** | **Working principle** |
| --- | --- | --- |
| cellTree | raw | Latent Dirichlet Allocation |
| kmeans | normalized | kmeans |
| Leiden | normalized | Community detection on shared nearest neighbor network |
| Monocle3 | normalized | Community detection on k nearest neighbor network |
| SC3 | normalized | Consensus clustering |

**Supplementary Table 4**: Summary of datasets.

| Dataset | Platform | Resolution | Number of spots or cells | Number of total genes | Ground Truth Labels |
| --- | --- | --- | --- | --- | --- |
| Mouse Brain Olfactory Bulb Dataset (replicate 1) | Visium | non-single-cell | 265 | 16573 | Obtained from original study: manual annotation based on cluster-specific differentially expressed marker genes. |
| HER2 Breast Cancer Dataset (sample A3) | Visium | non-single-cell | 343 | 14851 | Obtained from original study: manual annotation based on top, cluster-specific enriched pathways based on Gene Ontology—Biological Processes database. |
| HER2 Breast Cancer Dataset (sample B3) | Visium | non-single-cell | 262 | 14583 | Obtained from original study: manual annotation based on top, cluster-specific enriched pathways based on Gene Ontology—Biological Processes database. |
| HER2 Breast Cancer Dataset (sample B6) | Visium | non-single-cell | 239 | 14721 | Obtained from original study: manual annotation based on top, cluster-specific enriched pathways based on Gene Ontology—Biological Processes database. |
| HER2 Breast Cancer Dataset (sample D1) | Visium | non-single-cell | 306 | 15301 | Obtained from original study: manual annotation based on top, cluster-specific enriched pathways based on Gene Ontology—Biological Processes database. |
| HER2 Breast Cancer Dataset (sample D2) | Visium | non-single-cell | 302 | 14992 | Obtained from original study: manual annotation based on top, cluster-specific enriched pathways based on Gene Ontology—Biological Processes database. |
| HER2 Breast Cancer Dataset (sample D3) | Visium | non-single-cell | 300 | 15088 | Obtained from original study: manual annotation based on top, cluster-specific enriched pathways based on Gene Ontology—Biological Processes database. |
| HER2 Breast Cancer Dataset (sample D5) | Visium | non-single-cell | 304 | 15165 | Obtained from original study: manual annotation based on top, cluster-specific enriched pathways based on Gene Ontology—Biological Processes database. |
| HER2 Breast Cancer Dataset (sample E1) | Visium | non-single-cell | 586 | 14884 | Obtained from original study: manual annotation based on top, cluster-specific enriched pathways based on Gene Ontology—Biological Processes database. |
| Ovarian Cancer Dataset (sample B_GTFB1191) | Visium | non-single-cell | 2389 | 15508 | Obtained from original study: manual annotation based on cluster-specific GSEA. |
| Human Kidney Dataset (sample N7) | Xenium | single-cell | 394 | 345 | Obtained from original study: topological automatic cell type identification (TopACT) using multiscale gene expression data. |
| Human Kidney Dataset (sample O7) | Xenium | single-cell | 658 | 357 | Obtained from original study: topological automatic cell type identification (TopACT) using multiscale gene expression data. |
| Human Kidney Dataset (sample O8) | Xenium | single-cell | 1245 | 364 | Obtained from original study: topological automatic cell type identification (TopACT) using multiscale gene expression data. |
| Human Kidney Dataset (sample R7) | Xenium | single-cell | 2382 | 374 | Obtained from original study: topological automatic cell type identification (TopACT) using multiscale gene expression data. |
| Human Kidney Dataset (sample S7) | Xenium | single-cell | 1904 | 375 | Obtained from original study: topological automatic cell type identification (TopACT) using multiscale gene expression data. |
| Human Kidney Dataset (sample T7) | Xenium | single-cell | 1556 | 375 | Obtained from original study: topological automatic cell type identification (TopACT) using multiscale gene expression data. |
| Human Kidney Dataset (sample U7) | Xenium | single-cell | 988 | 370 | Obtained from original study: topological automatic cell type identification (TopACT) using multiscale gene expression data. |
| Mouse Brain ATN Dataset (sample S1 anterior) | Xenium | single-cell | 641 | 297 | Obtained from original study: manual annotation based on markers from matched scRNA-seq data. |
| Mouse Brain ATN Dataset (sample S2 interior) | Xenium | single-cell | 713 | 297 | Obtained from original study: manual annotation based on markers from matched scRNA-seq data. |
| Mouse Brain Hypothalamus Dataset (bregma: -140) | MERFISH | single-cell | 5024 | 152 | Obtained from original study: manual annotation based on matched scRNA-seq data. |
| Mouse Brain Hypothalamus Dataset (bregma: -190) | MERFISH | single-cell | 5193 | 151 | Obtained from original study: manual annotation based on matched scRNA-seq data. |
| Mouse Brain Hypothalamus Dataset (bregma: -240) | MERFISH | single-cell | 4926 | 152 | Obtained from original study: manual annotation based on matched scRNA-seq data. |
| Mouse Brain Hypothalamus Dataset (bregma: -290) | MERFISH | single-cell | 4913 | 151 | Obtained from original study: manual annotation based on matched scRNA-seq data. |
| Mouse Brain Hypothalamus Dataset (bregma: -40) | MERFISH | single-cell | 4852 | 153 | Obtained from original study: manual annotation based on matched scRNA-seq data. |
| Mouse Brain Hypothalamus Dataset (bregma: -90) | MERFISH | single-cell | 4984 | 153 | Obtained from original study: manual annotation based on matched scRNA-seq data. |
| Mouse Brain Hypothalamus Dataset (bregma: 10) | MERFISH | single-cell | 4985 | 152 | Obtained from original study: manual annotation based on matched scRNA-seq data. |
| Mouse Brain Hypothalamus Dataset (bregma: 110) | MERFISH | single-cell | 4699 | 151 | Obtained from original study: manual annotation based on matched scRNA-seq data. |
| Mouse Brain Hypothalamus Dataset (bregma: 160) | MERFISH | single-cell | 4749 | 150 | Obtained from original study: manual annotation based on matched scRNA-seq data. |
| Mouse Brain Hypothalamus Dataset (bregma: 210) | MERFISH | single-cell | 4374 | 148 | Obtained from original study: manual annotation based on matched scRNA-seq data. |
| Mouse Brain Hypothalamus Dataset (bregma: 260) | MERFISH | single-cell | 4417 | 148 | Obtained from original study: manual annotation based on matched scRNA-seq data. |
| Mouse Brain Hypothalamus Dataset (bregma: 60) | MERFISH | single-cell | 4698 | 152 | Obtained from original study: manual annotation based on matched scRNA-seq data. |
| Human Lung NSCLC Dataset (patient 5, Rep 1, FOV 1) | cosMx | single-cell | 1803 | 960 | Obtained from original study: cell type annotated through morphology-based cell segmentation (Insitutype). |
| Human Lung NSCLC Dataset (patient 5, Rep 1, FOV 23) | cosMx | single-cell | 2436 | 960 | Obtained from original study: cell type annotated through morphology-based cell segmentation (Insitutype). |
| Human Lung NSCLC Dataset (patient 5, Rep 1, FOV 25) | cosMx | single-cell | 3589 | 960 | Obtained from original study: cell type annotated through morphology-based cell segmentation (Insitutype). |
| Human Lung NSCLC Dataset (patient 5, Rep 2, FOV 14) | cosMx | single-cell | 2067 | 960 | Obtained from original study: cell type annotated through morphology-based cell segmentation (Insitutype). |
| Human Lung NSCLC Dataset (patient 5, Rep 2, FOV 18) | cosMx | single-cell | 2759 | 960 | Obtained from original study: cell type annotated through morphology-based cell segmentation (Insitutype). |
| Human Lung NSCLC Dataset (patient 5, Rep 2, FOV 20) | cosMx | single-cell | 3627 | 960 | Obtained from original study: cell type annotated through morphology-based cell segmentation (Insitutype). |
| Human Lung NSCLC Dataset (patient 5, Rep 2, FOV 21) | cosMx | single-cell | 2489 | 960 | Obtained from original study: cell type annotated through morphology-based cell segmentation (Insitutype). |
| Human Lung NSCLC Dataset (patient 5, Rep 2, FOV 24) | cosMx | single-cell | 2055 | 960 | Obtained from original study: cell type annotated through morphology-based cell segmentation (Insitutype). |
| Human Lung NSCLC Dataset (patient 5, Rep 2, FOV 7) | cosMx | single-cell | 3215 | 960 | Obtained from original study: cell type annotated through morphology-based cell segmentation (Insitutype). |
| Human Lung NSCLC Dataset (patient 5, Rep 3, FOV 12) | cosMx | single-cell | 2340 | 960 | Obtained from original study: cell type annotated through morphology-based cell segmentation (Insitutype). |
| Human Lung NSCLC Dataset (patient 5, Rep 3, FOV 13) | cosMx | single-cell | 2016 | 960 | Obtained from original study: cell type annotated through morphology-based cell segmentation (Insitutype). |
| Human Lung NSCLC Dataset (patient 5, Rep 3, FOV 14) | cosMx | single-cell | 2126 | 960 | Obtained from original study: cell type annotated through morphology-based cell segmentation (Insitutype). |
| Human Lung NSCLC Dataset (patient 5, Rep 3, FOV 15) | cosMx | single-cell | 1967 | 960 | Obtained from original study: cell type annotated through morphology-based cell segmentation (Insitutype). |
| Human Lung NSCLC Dataset (patient 5, Rep 3, FOV 19) | cosMx | single-cell | 2099 | 960 | Obtained from original study: cell type annotated through morphology-based cell segmentation (Insitutype). |
| Human Lung NSCLC Dataset (patient 5, Rep 3, FOV 22) | cosMx | single-cell | 1971 | 960 | Obtained from original study: cell type annotated through morphology-based cell segmentation (Insitutype). |
| Human Lung NSCLC Dataset (patient 5, Rep 3, FOV 24) | cosMx | single-cell | 2180 | 960 | Obtained from original study: cell type annotated through morphology-based cell segmentation (Insitutype). |
| Human Lung NSCLC Dataset (patient 5, Rep 3, FOV 25) | cosMx | single-cell | 3466 | 960 | Obtained from original study: cell type annotated through morphology-based cell segmentation (Insitutype). |
| Human Lung NSCLC Dataset (patient 5, Rep 3, FOV 27) | cosMx | single-cell | 3239 | 960 | Obtained from original study: cell type annotated through morphology-based cell segmentation (Insitutype). |
| Human Lung NSCLC Dataset (patient 5, Rep 3, FOV 28) | cosMx | single-cell | 2643 | 960 | Obtained from original study: cell type annotated through morphology-based cell segmentation (Insitutype). |
| Human Lung NSCLC Dataset (patient 5, Rep 3, FOV 30) | cosMx | single-cell | 3892 | 960 | Obtained from original study: cell type annotated through morphology-based cell segmentation (Insitutype). |

**Supplementary Table 5**: Paired effect sizes and p-values of Wilcoxon test for Leiden clustering.

| **Metric** | **Platform** | **Gene sets** | **Effect size** | **p-value** |
| --- | --- | --- | --- | --- |
| AMI | MERFISH | Concatenation v.s. SV genes | 1.39e-04 | 9.66e-01 |
|  |  | Concatenation v.s. HV genes | 1.84e-01 | 4.886e-04 |
|  | cosMx | Concatenation v.s. SV genes | 3.43e-02 | 2.10e-04 |
|  |  | Concatenation v.s. HV genes | 6.04e-02 | 8.20e-05 |
|  | Xenium | Concatenation v.s. SV genes | 3.23e-03 | 5.47e-01 |
|  |  | Concatenation v.s. HV genes | 2.4e-02 | 7.81e-03 |
|  | Visium | Concatenation v.s. SV genes | 9.33e-03 | 4.88e-02 |
|  |  | Concatenation v.s. HV genes | 3.17e-02 | 9.77e-03 |
| Weighted F1 | MERFISH | Concatenation v.s. SV genes | 5.43e-04 | 4.65e-01 |
|  |  | Concatenation v.s. HV genes | 1.58e-01 | 9.77e-04 |
|  | cosMx | Concatenation v.s. SV genes | 7.26e-02 | 1.02e-03 |
|  |  | Concatenation v.s. HV genes | 6.66e-02 | 6.39e-03 |
|  | Xenium | Concatenation v.s. SV genes | 3.35e-03 | 1.95e-01 |
|  |  | Concatenation v.s. HV genes | 1.81e-02 | 3.01e-01 |
|  | Visium | Concatenation v.s. SV genes | 2e-02 | 1.05e-01 |
|  |  | Concatenation v.s. HV genes | 7.01e-03 | 6.25e-01 |
| Pearson Gamma | MERFISH | Concatenation v.s. SV genes | 6.98e-03 | 5.37e-02 |
|  |  | Concatenation v.s. HV genes | 1.67e-01 | 4.880-04 |
|  | cosMx | Concatenation v.s. SV genes | 2.17e-02 | 7.59e-02 |
|  |  | Concatenation v.s. HV genes | 3.18e-02 | 7.59e-02 |
|  | Xenium | Concatenation v.s. SV genes | -1.35e-03 | 8.20e-01 |
|  |  | Concatenation v.s. HV genes | 1.81e-02 | 2.50e-01 |
|  | Visium | Concatenation v.s. SV genes | -2.03e-03 | 6.95e-01 |
|  |  | Concatenation v.s. HV genes | 2.97e-03 | 4.32e-01 |
| Spatial Concordance | MERFISH | Concatenation v.s. SV genes | 3.30e-03 | 8.30e-02 |
|  |  | Concatenation v.s. HV genes | 1.85e-01 | 4.88e-04 |
|  | cosMx | Concatenation v.s. SV genes | 3.92e-02 | 5.86e-04 |
|  |  | Concatenation v.s. HV genes | 7.42e-02 | 1.02e-03 |
|  | Xenium | Concatenation v.s. SV genes | 7.21e-03 | 4.61e-01 |
|  |  | Concatenation v.s. HV genes | 1.25e-02 | 4.96e-01 |
|  | Visium | Concatenation v.s. SV genes | 1.26e-02 | 1.05e-01 |
|  |  | Concatenation v.s. HV genes | 1.29e-02 | 5.57e-01 |
| Mean Spatial AMI | MERFISH | Concatenation v.s. SV genes | 3.28e-03 | 8.30e-02 |
|  |  | Concatenation v.s. HV genes | 2.00e-01 | 4.88e-04 |
|  | cosMx | Concatenation v.s. SV genes | 4.34e-02 | 1.34e-04 |
|  |  | Concatenation v.s. HV genes | 7.73e-02 | 1.21e-03 |
|  | Xenium | Concatenation v.s. SV genes | 4.39e-03 | 4.61e-01 |
|  |  | Concatenation v.s. HV genes | 1.62e-02 | 5.70e-01 |
|  | Visium | Concatenation v.s. SV genes | 3.51e-03 | 6.95e-01 |
|  |  | Concatenation v.s. HV genes | 1.06e-02 | 5.57e-01 |
